# Identification and fine mapping of a new gene, *BPH31* conferring resistance to brown planthopper biotype 4 of India to improve rice, *Oryza sativa* L

**DOI:** 10.1186/s12284-017-0178-x

**Published:** 2017-08-31

**Authors:** G. D. Prahalada, N. Shivakumar, H. C. Lohithaswa, D. K. Sidde Gowda, G. Ramkumar, Sung-Ryul Kim, C. Ramachandra, Shailaja Hittalmani, Trilochan Mohapatra, Kshirod K. Jena

**Affiliations:** 1 Plant Breeding Division, International Rice Research Institute, DAPO Box 7777, Metro Manila, Philippines; 2 Zonal Agricultural Research Station, VC Farm, Mandya, Karnataka India; 3 College of Agriculture, Mandya, Karnataka India; 4 University of Agricultural Sciences, GKVK, Bangalore, Karnataka India; 5 Indian Council of Agricultural Research, New Delhi, India

**Keywords:** Host-plant resistance, *BPH31*, InDel marker, Marker-assisted selection, Resistance mechanism

## Abstract

**Background:**

Rice (*Oryza sativa* L*.*) is the staple food for more than 3.5 billion people, mainly in Asia. Brown planthopper (BPH) is one of the most destructive insect pests of rice that limits rice production. Host-plant resistance is one of the most efficient ways to overcome BPH damage to the rice crop.

**Results:**

BPH bioassay studies from 2009 to 2015 conducted in India and at the International Rice Research Institute (IRRI), Philippines, revealed that the cultivar CR2711–76 developed at the National Rice Research Institute (NRRI), Cuttack, India, showed stable and broad-spectrum resistance to several BPH populations of the Philippines and BPH biotype 4 of India. Genetic analysis and fine mapping confirmed the presence of a single dominant gene, *BPH31,* in CR2711–76 conferring BPH resistance. The *BPH31* gene was located on the long arm of chromosome 3 within an interval of 475 kb between the markers PA26 and RM2334. Bioassay analysis of the *BPH31* gene in CR2711–76 was carried out against BPH populations of the Philippines. The results from bioassay revealed that CR2711–76 possesses three different mechanisms of resistance: antibiosis, antixenosis, and tolerance. The effectiveness of flanking markers was tested in a segregating population and the InDel type markers PA26 and RM2334 showed high co-segregation with the resistance phenotype. Foreground and background analysis by tightly linked markers as well as using the Infinium 6 K SNP chip respectively were applied for transferring the *BPH31* gene into an indica variety, Jaya. The improved *BPH31-*derived Jaya lines showed strong resistance to BPH biotypes of India and the Philippines.

**Conclusion:**

The new *BPH31* gene can be used in BPH resistance breeding programs on the Indian subcontinent. The tightly linked DNA markers identified in the study have proved their effectiveness and can be utilized in BPH resistance breeding in rice.

**Electronic supplementary material:**

The online version of this article (doi:10.1186/s12284-017-0178-x) contains supplementary material, which is available to authorized users.

## Background

Rice is one of the most important and staple food crop that feed more than half of the world’s population. However, rice production is severely affected by biotic and abiotic stresses. About 52% of the total global rice production is lost annually because of the damage caused by biotic factors, of which nearly 21% is attributed to the attack of insect pests (Khush 1979; Sogawa et al. 2003). Among the insect pests, the brown planthopper (BPH) *Nilaparvata lugens* (Stal) is one of the most destructive monophagous insect pests of rice throughout Asian rice-growing countries. In recent years, BPH infestations have intensified across Asia, causing significant yield losses (Normile 2008). BPH not only causes direct damage to the rice crop by sucking plant sap, often resulting in “hopper burn,” but it can also cause indirect damage by transmitting virus diseases such as rice grassy stunt and ragged stunt (Cabauatan et al. 2009). Application of pesticides is the most common practice to control BPH. However, this method is costly and hazardous to the environment and health. Moreover, application of pesticide often leads to a resurgence of the pest population and affects natural BPH predators (Tanaka et al. 2000). The use of host-plant resistance is considered as one of the most economical and effective measures for BPH management (Ramkumar et al. 2016).

Considerable effort has been made in the search for rice genes conferring host-plant resistance to BPH. Thirty BPH resistance genes have been identified (Deen et al. 2017) and mapped to six of the 12 chromosomes (2, 3, 4, 6, 11, and 12) of rice (Cheng et al. 2013). Among those, only 17 genes (*BPH1*, *BPH2*, *BPH6*, *BPH9*, *BPH12*, *BPH14*, *BPH15*, *BPH17, BPH18*, *BPH19*, *BPH25*, *BPH26*, *BPH27*, *BPH28, BPH29*, *BPH30* and *BPH32*) have been fine-mapped (Cha et al. 2008; Sun et al. 2006; Zhao et al. 2016; Qiu et al. 2012; Du et al. 2009; Lv et al. 2014; Liu et al. 2015; Jena et al. 2006; Chen et al. 2006; Myint et al. 2012; Tamura et al. 2014; Huang et al. 2013; Wu et al. 2014; Wang et al. 2015; Ren et al. 2016). However, only seven genes (*BPH14, BPH17, BPH18, BPH26, BPH29, BPH9* and *BPH32*) have been cloned and characterized (Du et al. 2009; Liu et al. 2015; Tamura et al. 2014; Wang et al. 2015; Ji et al. 2016; Zhao et al. 2016; Ren et al. 2016). Unfortunately, most of the identified resistance genes are biotype/population specific and do not provide strong resistance to different BPH biotypes/populations. Hence, it is imperative to identify new broad-spectrum BPH resistance genes and transfer them into elite BPH-susceptible cultivars for stable rice production.

Plants can employ various resistance mechanisms to reduce insect damage in nature. Plant resistance to insects is generally differentiated in (1) antibiosis: a quality that reduces insect survival, growth rate, or reproduction following the ingestion of host tissue; (2) antixenosis: a quality that repels or disturbs insects, causing a reduction in colonization or oviposition; and (3) tolerance: a capacity to produce a crop of high quality and yield despite insect infestation (Alam and Cohen 1998). BPH resistance genes confer their resistance through one or a combination of the above-mentioned defense mechanisms. Understanding the mode of gene action leads to better BPH management practices. However, the information on resistance mechanism of most of the identified BPH resistance genes is limited. Therefore, it is necessary to analyze the mechanisms of resistance in resistant cultivars carrying BPH resistance genes, which should favor the BPH resistance breeding programs in rice.

BPH populations have been categorized into four different biotypes (Khush et al. 1985; Brar et al. 2010). The population in East and Southeast Asia is reported as biotype 1, whereas biotype 2 originated in Indonesia and Vietnam as a dominant biotype (Pathak and Khush, 1979). Biotype 3 was produced in the laboratory at the International Rice Research Institute (IRRI) (Pathak and Khush 1979), whereas biotype 4 is found only in South Asia, especially on the Indian subcontinent. Although there are at least 30 reported BPH resistance genes, only a few genes showed their resistance potential and broad spectrum resistace to Indian biotype 4 (Deen et al. 2010; Horgan et al. 2015). In addition, most of the resistance genes were identified from rice cultivars that had inferior grain quality and agronomic traits. On the other hand, most of the rice varieties that have superior yield and agronomic traits are generally susceptible to BPH infestation. Hence, to obtain the real and maximum benefits of host-plant resistance genes, the identified resistance genes have to be introgressed into elite rice varieties without affecting their yield-associated traits and superior grain quality traits.

In order to address the constraints mentioned above, the present study was carried out with the follwoing objectives: (1) identification and genetic analysis of BPH resistance patterns in the resistant cultivar CR2711–76; (2) fine mapping of a major BPH resistance gene, *BPH31*; (3) incorporation of the *BPH31* gene into an elite *O. sativa* subspecies indica cultivar, Jaya; and (4) exploring the resistance mechanisms of the new BPH resistance gene.

## Results

### BPH bioassay and genetic analysis of the parental lines and mapping population

To evaluate the resistance strength and spectrum of the donor variety CR2711–76, we conducted the BPH bioassay using different BPH populations of the Philippines representing three biotypes, biotype 1, 2 and 3 duirng 2013–2015 and BPH biotype 4 in 2009, 2010, 2011, 2013, and 2014 in India. For comparisons with the existing popular BPH resistance sources, Rathu Heenati (*BPH3* and *BPH17* donor) and PTB-33 (*BPH2* and *BPH3* donor) were included. The variety CR2711–76 showed strong resistance, as much as the resistant check lines Rathu Heenati and PTB-33, against both BPH biotype 4 and Laguna BPH populations (Table 1). These results indicated that the new BPH resistance source, CR2711–76, had strong and broad-spectrum resistance. The F_1_ plants from the cross between Jaya and CR2711–76 showed strong resistance to both BPH biotype 4 and Laguna BPH populations suggesting that the resistance was conferred by a dominant genetic factor (Table 1).

**Table 1:** BPH reaction score of parents, F_1_, and check varieties against biotype 4 and Laguna BPH colony

Genotypes^a^	Number of seedlings tested	Average resistance score	Significance^b^
Reaction to BPH biotype 4 (2009–2014)			
CR2711–76	201	2.33	a
Jaya	207	7.99	b
F_1_ (Jaya/CR2711–76)	52	1.33	a
PTB-33	206	4.03	a
Reaction to Laguna BPH colony (2013–2015)			
CR2711–76	199	3.87	b
Jaya	176	8.91	a
F_1_ (Jaya/CR2711–76)	24	2.88	b
Rathu Heenati	210	3.11	b
TN-1	272	9.01	a

^a^ TN-1 and Jaya were used as susceptible checks and PTB-33 and Rathu Heenati were used as resistant checks

^b^ Least significant difference test at α <0.01

The genetic analysis was performed using approximately 3800 F_3_ plants derived from 151 F_2_ plants, total of 39 F_3_ families were susceptible, 85 families were segregating, and 27 families were resistant. The F_2_ population showed a 1:2:1 (χ^2^_(0.11)_ = 4.29) genotypic segregation ratio and 3:1 (124R:27S) phenotypic segregation ratio. In addition to this, mode of inheritance was also analysed using another set of F_2_, BC_1_F_1_ and BC_2_F_2_ populations consisting of 168, 101 and 249 individuals and the segregation ratio of 3:1, 1:1 and 3:1 (R: S) respectively (Table 2) was obtained. The segregation pattern from these results indicated that BPH resistance derived from the donor CR2711–76 was controlled by a single dominant gene.

**Table 2:** The genetic analysis of different maping populations derived from Jaya × CR2711–76 cross evaluated against BPH

Mapping population type	Resistant plants (No.)	Susceptible plants (No.)	Total plants (No.)	Segregation (R:S)	χ^2^	*α* value
^a^ F_2_ (1st set)	123	28	151	3:01	3.35	0.06
^a^ F_2_ (2nd set)	131	37	168	3:01	0.79	0.37
^a^ BC_1_F_1_	55	46	101	1:01	0.8	0.37
^b^ BC_2_F_2_	181	68	249	3:01	0.7	0.4

^a^ BPH biotype 4

^b^ Laguna BPH population

### Mechanisms of BPH resistance

**Fig. 1:**
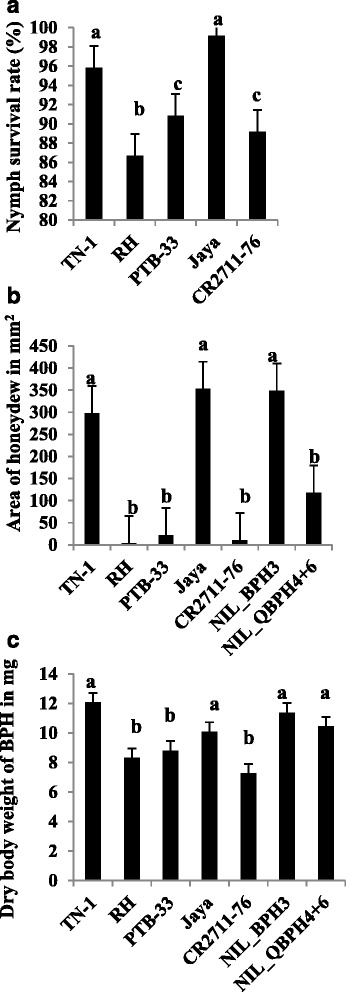
Antibiosis mechanism tests. **a** Nymph survival rate of different test lines. **b** Area of honeydew secretion as the parameter of antibiosis on feeding rate. **c** Dried body weight of BPH female gravids as the parameter of antibiosis on feeding rate. Error bar represents the standard error of different replications

To reveal the resistance mechanisms such as antibiosis, antixenosis, and tolerance of BPH resistant donor, CR2711–76, several experiments were conducted along with Jaya, and several known BPH-resistant lines. Firstly, the antibiosis mechanism was assessed through nymph survival data. The average nymph survival rate was the highest in Jaya (98.66%), followed by TN-1 (95.45%). The lowest nymph survival was observed in CR2711–76 (87.92%) and it was in the same group as the BPH-resistant lines PTB-33 (85.70%) by DMRT statistical analysis (Fig. 1a). This result indicated that CR2711–76 possessed the antibiosis resistance mechanism. In addition, the area of honeydew secretion as feeding rate was also measured to study the antibiosis on feeding rate mechanism. Our results showed that the average area of honeydew secreted for CR2711–76 (10.33 mm^2^) was significantly lower than the Jaya (352.71 mm^2^) (Fig. 1b). We also measured the dry weight of adult female gravids as a feeding rate indicator. Two independent experiments on dry body weight of adult female gravids of Laguna BPH populations revealed that the lowest body weight of 7.25 mg was observed on the BPH female gravids, which fed on cultivar CR2711–76, followed by Rathu Heenati and PTB-33 with 8.31 mg and 8.80 mg, respectively. The highest body weight was recorded in the female gravids that fed on TN-1, followed by near isogenic lines (NIL), NIL*-BPH3,* NIL-*QBPH4* and *QBPH6* with 12.07 mg, 11.37 mg, and 10.44 mg, respectively. BPH fed more on TN-1, NIL*-BPH3,* NIL-*QBPH4,* and *QBPH6* than on CR2711–76, Rathu Heenati, and PTB-33 (Fig. 1c). These three different assays (antibiosis on nymph survival rate, feeding rate on area of honey dew and BPH body weight) strongly support that CR2711–76 has the antibiosis mechanism for BPH resistance.

**Fig. 2:**
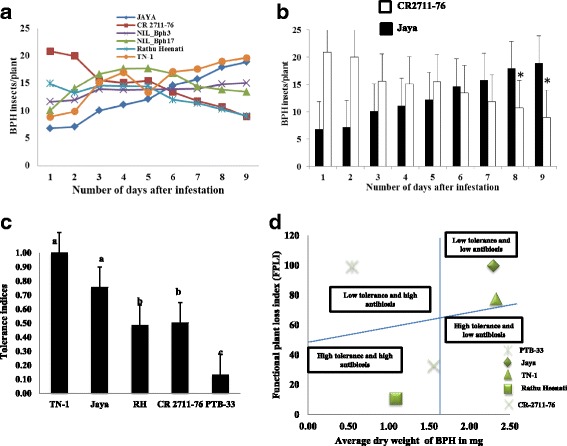
Antixenosis and tolerance mechanism tests. **a** Non-preference behavior of BPH adults for different test genotypes at the different days after infestation with each colored lines indicating different genotypes. **b** Non-preference behavior of BPH nymphs for parental lines, Jaya and CR2711–76; error bars indicate the standard error of standard deviation of three replications. **c** Tolerance indices of test lines. **d** FPLI graph showing the differentiation of antibiosis and tolerance mechanisms of resistance to BPH. Letters *a,b,c* indicate DMRT significance values (α = 0.01)

To test the antixenosis mechanism, the number of BPH insects was monitored on the different genotypes for 9 days after BPH infestation, when there was 90% TN-1 wilted. There was a significant decrease in the number of insects in the resistant lines, CR2711–76 (20.85 to 8.97) and Rathu Heenati (14.96 to 9.08) while BPH nymphs significantly increased in the susceptible lines, Jaya (6.83 to 18.88) and TN-1 (8.89 to 19.67). On the ninth day after BPH infestation, significant difference in the total load of BPH nymphs on the CR2711–76 (8.97) and Jaya (18.88) was observed (Additional file 8: Figure S8). This result clearly indicated that, CR2711–76 possess the antixenosis mechanism of resistance (Fig. 2a and b). However, for NIL*-BPH3* and NIL*-BPH17*, no significant difference was observed with respect to the number of BPH per hill for 9 days.

To analyze the tolerance mechanism of the cultivars, the functional plant loss index (FPLI) and tolerance index (TI) were calculated. TI was observed to be the lowest in PTB-33, followed by Rathu Heenati and CR2711–76, with TI values of 0.13, 0.48, and 0.50, respectively. No significant difference was observed among the TI values of Jaya and TN-1, indicating that they were on par with the tolerance mechanism (Fig. 2c). To analyze FPLI, a regression line was generated from the regression of FPLI on the dry weight of BPH and the mean dry weight of BPH divided the graph into four quadrants (sectors). As expected, Jaya and TN-1, with high body weight of BPH and high FPLI values, congregated in the low tolerance and low antibiosis (susceptible) quadrant. PTB-33, with high FPLI value and low BPH body weight, congregated in the high antibiosis and low tolerance quadrant. However, Rathu Heenati and CR2711–76, with lower BPH body weight and low FPLI, congregated in the high tolerance and high antibiosis quadrant. This experiment of tolerance segregated the resistant varieties with different mechanisms of resistance (Fig. 2d). From this experiment, it was evident that CR2711–76 possesses a tolerance mechanism to provide resistance for a long period of time (Table 1).

### Broad-spectrum resistance of the CR2711–76

The resistance spectrum of CR2711–76 was tested by measuring the honeydew secretion area against the three different BPH populations that were collected in the three different provinces (Isabela, Bicol, and Nueva Ecija) in the Philippines. For the Isabela BPH populations, the highest and the lowest honeydew secretion area were observed on TN-1 (846.41 mm^2^) and CR2711–76 (84.69 mm^2^), respectively (Additional file 1: Figure S1a)**.** For Bicol BPH populations, the highest honeydew secretion area was noticed in TN-1 (536.74 mm^2^), followed by Jaya (502.61 mm^2^), whereas the lowest honeydew secretion area was observed in CR2711–76 (103.27 mm^2^) (Additional file 1: Figure S1b). Interestingly, honeydew secretion was completely absent in CR2711–76 and F_1_ generated from the cross of Jaya × CR2711–76 when Nueva Ecija BPH adults fed on this line (Additional file 1: Figure S1c; Additional file 2: Figure S2). In addition to this, antibiosis (seedling survival rate) of parental lines and F_1_s was also carried out to study the broad spectrum resistance of CR2711–76 in both India and the Philippines respectively. The significant difference in resistance score between CR2711–76 (resistant; 2.33 and 3.87) and Jaya (susceptible; 7.99 and 8.91) was observed when they were screened for both Indian and Philippines BPH colony during 2009–2014 and 2013–2015 respectively (Table 1). This result of antibiosis reaction of CR2711–76 against different BPH populations of the Philippines and India strongly support that, it possess broad-spectrum resistance.

### Inheritance of antibiosis on feeding rate

The inheritance of the resistance gene derived from CR2711–76 for BPH colonies of Laguna and Nueva Ecija was studied by assessing the antibiosis on feeding rate. The area of honeydew secreted was 31.39 mm^2^ after Laguna BPH colony fed on CR2711–76. It was smallest (4.78 mm^2^) and completely absent after Laguna and Nueva Ecija BPH populations fed on F_1_ plants respectively (Additional file 1: Figure S1d). This result showed that the BPH resistance is a dominance type of genetic factor*.*

### Linkage map construction and localization of a resistance gene

To identify the BPH resistance locus of the BPH resistant donor line, CR2711–76, PCR genotyping was performed using 151 F_2_ genomic DNAs with 107 polymorphic markers distributed over the entire rice genome. The mapping population was successively genotyped with all the 107 anchored markers, and a linkage map was constructed (Additional file 3: Figure S3). All the anchored markers on the framework map (linkage map skeleton) were tested for linkage relationships and a BPH resistance factor was initially localized on the long arm of chromosome 3 between the SSR markers RM251 and RM2334 through QTL analysis using QTL IciMapping software (Additional file 4: Figure S4a) spanning a region of 24.30 cM under extreme linkage criteria with a minimum LOD score of 11 at 1000 permutations. Only a single major locus from CR2711–76 present on the chromosome 3 was identified throughout 12 chromosomes which was consistent with our previous genetic analysis data suggesting three different mechanisms of resistance. The result was validated using WinQTL Cartographer and a similar result was retrieved (Additional file 4: Figure S4b). The putatively identified new resistance gene was named as *BPH31* (Additional file 10: Figure S10).

### Fine mapping and candidate gene identification at the *BPH31* locus

**Fig. 3:**
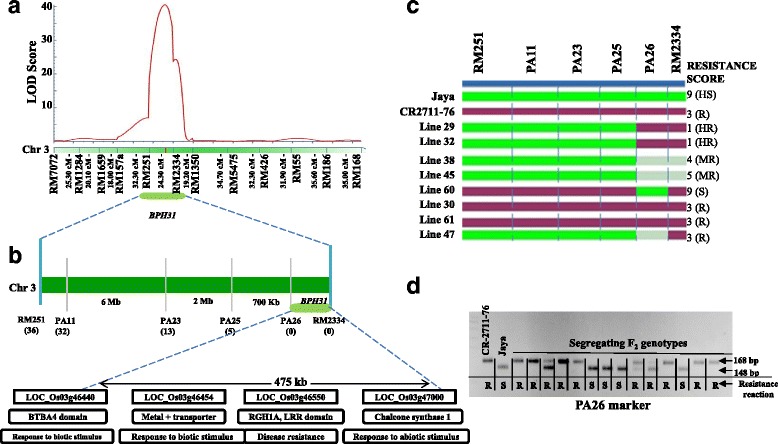
Primary and fine mapping of *BPH31* gene locus. **a** The primary map of *BPH31* using 151 F_2:3_ lines. **b** Physical and fine map of *BPH31* locus; g*ray vertical bars* indicate newly designed polymorphic InDel markers for fine mapping. **c** Molecular marker genotypes and phenotypes of recombinants. The g*reen, light red,* and *gray bars* denote the marker genotypes of Jaya homozygotes, CR2711–76 homozygotes, and their heterozygotes, respectively. **d** Segregation pattern of the PA26 InDel marker with BPH resistance reaction. R and S indicate resistance and susceptible reaction of BPH respectively

For fine mapping of the *BPH31* locus, 27 InDel markers were newly designed based on primary mapping results (Additional file 11: Table S1) and tested in the parents. Four markers showed polymorphism and these InDel markers were used to screen the same F_2_ population with 151 individuals and linkage analysis was further carried out. Finally, the putative resistance gene, *BPH31,* was located between the markers PA26 and RM2334 with a LOD score of 47.64 and 79.8% phenotypic variance. The physical size of the genetic locus between markers PA26 and RM2334 was 475 kb based on the Nipponbare reference genome in the TIGR database (Fig. 3a-d).

Genome annotation data of the fine-mapped *BPH31* locus (475 kb region) contained minimum of 42 candidate genes (Additional file 12: Table S2). We examined the putative functions of all 42 candidate genes using the BPLAST-P tool and it was revealed that three genes (*LOC_Os03g46440*, *LOC_Os03g46454,* and *LOC_Os03g46550*) might be associated with biotic stress stimulus. Among the three putative candidate genes, *LOC_Os03g46550* encodes LRR domain protein, which is found in many defense-related genes. In addition, in silico gene expression analysis in the Rice oligo nucleotide array and RiceXPro data base showed that *LOC_Os03g46550* gene preferentially expressed in the third- to fourth-leaf stage (Additional file 5: Figure S5a-c), when maximum infestation of BPH insect was observed.

### Validation of *BPH31* flanking marker for MAS

The efficacy of the markers within the primary mapped locus was tested using the BC_2_F_2_ population derived from the cross Jaya × CR2711–76. This was performed by genotyping (using the flanking markers of *BPH31*) and phenotyping the homo and heterozygous plants derived BC_2_F_2_ progenies. As expected, homozygous recessive BC_2_F_2_ plants showed a high susceptibility reaction to BPH while BC_2_F_2_ plants showed segregation in the BPH resistance reaction (Additional file 6: Figure S6). The seedling survival rate and SES score of 149 BC_2_F_2_ plants and flanking markers of fine-mapped *BPH31* genotype data were compared to analyze the percent co-segregation. The markers PA25, PA26 and RM2334 were showed high co-segregation with the BPH reaction. Among these markers, PA26 and RM2334 showed highest co-segregation. Hence, these markers were considered as tightly linked markers and used for introgressing *BPH31* in the background of Jaya.

### Marker-assisted introgression of *BPH31*

Based on the genotype of flanking markers, RM251, PA26, and RM2334 of the *BPH31* locus, 101 BC_1_F_1_ individuals were subjected to foreground selection. Of the 101 BC_1_F_1_s, 41 BC_1_F_1_ plants were found to have *BPH31,* whereas 60 individuals were negative for the presence of the *BPH31* gene. These 41 BC_1_F_1_ plants were selected for background analysis and phenotypic selection for improving Jaya.

**Fig. 4:**
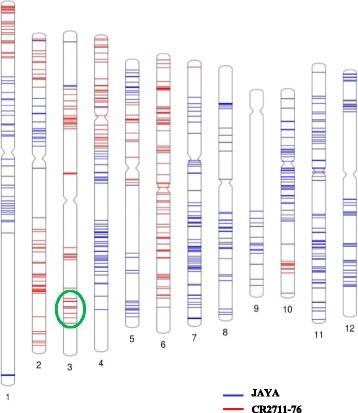
Graphical genotype map of the selected BC_2_F_1_ plant. The map was constructed based on the genotype data of the 575 polymorphic SNP markers on Infinium 6K SNP chip. Numbers below each chromosome indicate the respective chromosome number. *Blue* and *red lines* indicate the recurrent (Jaya) and the donor (CR2711–76) allele, respectively. The *BPH31* segment introgression is highlighted by *green circle*

Selected individuals of BC_1_F_1_s with the *BPH31* resistance gene were backcrossed to recipeient cultivar, Jaya to produce BC_2_F_1_ plants. Background genotyping analysis by high-density SNP markers revealed a significant quantity of recurrent parent genome recovery and the *BPH31* introgression from the donor parent at its respective locus (Fig. 4). Phenotypic selection was also practiced to select genotypes with superior agronomic traits. The selected individuals were superior in agronomic traits such as larger panicle length (ranging from 18.40 cm to 26.50 cm) and higher grain yield per plant than the original recurrent parent, Jaya. However, the grain type was similar to Jaya (Table 3).

**Table 3:** List of genotypes with agronomically important traits

No.	Genotypes	Grain type	Panicle length (cm)	Grain yield per plant (g)
1	Jaya	long bold	22.80^a^	17.10^a^
2	CR2711–76	short bold	19.70^a^	13.06^a^
3	BC1F1–3-BC2F1–1–1-110	short bold	20.50	14.36
4	BC1F1–5-BC2F1–2–1-133	long bold	25.50	19.15
5	BC1F1–32-BC2F1–7–1-167	medium bold	24.30	14.40
6	BC1F1–42-BC2F1–13–1-250	medium bold	21.70	13.88
7	BC1F1–32-BC2F1–7–12–166	long bold	22.00	18.66
8	BC1F1–54-BC2F1–17–11–291	short bold	18.40	16.76
9	BC1F1–40-BC2F1–11–2-215	short bold	22.50	9.11
10	BC1F1–35-BC2F1–8–7-176	long bold	26.50	18.50
		Mean	22.39	15.50
		Standard error	0.80	0.99
		Range	8.10	10.04
		Minimum	18.40	9.11
		Maximum	26.50	19.15
		Confidence level (95.0%)	1.81	2.24

^a^ Indicates average values of each observation

## Discussion

BPH is the most devastating pest of the rice crop and severe infestation causes “hopper burn” symptom in rice fields, resulting in complete death of rice plants. This pest causes serious problems in rice production worldwide, especially in Asia. Among the four classified different biotypes of BPH, biotype 4 is the most damaging and prevalent insect pest in South Asian countries, especially on the Indian subcontinent. Only a few resistance genes are available for providing resistance against this biotype 4. Hence, it is essential to identify and characterize new BPH resistance genes for stable rice production. Because only the identification of the sources of resistance genes is not sufficient to use in breeding, it is necessary to locate the gene and identify the tightly linked molecular markers for the gene. Additionally, BPH resistance genes act in different ways to keep the plant healthier. In a suitable BPH resistance breeding program, it will be necessary to know the mode of gene action to pyramid the right combinations of genes. Using location/linked markers and considering their mode of action, the resistance gene has to be introgressed into BPH-susceptible elite rice cultivars for sustainable rice production. In this study, we identified a strong broad-spectrum BPH resistance gene, *BPH31* and fine-mapped the gene on the long arm of chromosome 3. The analysis on the mode of gene action revealed interesting results: that the gene had all the three different kinds of resistance mechanisms (antibiosis, antixenosis, and tolerance) against BPH insects. To prove the applicability of the new *BPH31* gene in a BPH resistance breeding program, the gene was successfully introgressed into an elite indica cultivar (Jaya) using the tightly linked InDel marker PA26 and RM2334.

The BPH resistance source was an indica rice cultivar, CR2711–76. This cultivar was selected as a donor for the BPH resistance gene identification program as it had been providing resistance for the last seven consecutive years in our evaluation experiments in the region, which was prone to severe BPH infestation. Irrespective of the BPH infestation pressure, CR2711–76 provides strong BPH resistance, which indicates that it possesses a strong and broad-spectrum BPH resistance gene.

Understanding the mechanisms underlying resistance to BPH is essential for developing appropriate breeding strategies (Qiu et al. 2012). Hence, we have also explored all three kinds of mechanisms (antibiosis, antixenosis, and tolerance) underlying BPH resistance.

Antibiosis on nymph survival studies revealed that BPH nymphs feeding on CR2711–76 showed a high mortality rate in contrast to Jaya and susceptible check TN-1. The DMRT results clearly indicated that CR2711–76 possesses antibiosis on nymph survival against Laguna BPH populations compared to Jaya with low nymphal mortality. Hence, CR2711–76 can be used for developing a cultivar that can reduce BPH load in the field at the nymphal stage of insect attack. The area of honeydew secreted and the dry body weights of BPH female gravids are the parameters for measuring the antibiosis on feeding rate. The area of honeydew secreted when BPH feeds on Rathu Heenati was the lowest, followed by CR2711–76 and PTB-33, in the first experiment. This experiment revealed that BPH female gravids fed very low phloem sap or no phloem sap from the resistant cultivars (CR2711–76, Rathu Heenati, and PTB-33) compared to TN-1 and Jaya, on which they feed more phloem sap, and therefore they exuded more honeydew, which was witnessed as a blue color stain on the bromocresol green treated filter paper (Heinrichs et al. 1985). In addition, the difference in the feeding behavior of the Rathu Heenati and NIL-*BPH3* test lines was noticed. This could be because, Rathu Heenati also possess another major BPH resistance gene *BPH17* (Sun et al., 2005) and other BPH resistance QTLs, *qBPH3, qBPH4* and *qBPH10* (Acc.no. 11730; Sun et al. 2005) and other reason could be due to background effect of the NIL. Furthermore, the oven-dry body weight of BPH adults was also the lowest in CR2711–76, followed by Rathu Heenati and PTB-33, meaning that CR2711–76 allowed BPH female gravids to consume very low amounts of plant biomass, followed by Rathu Heenati and PTB-33 (Pathak and Heinrichs 1982). It should be noted that antibiosis on feeding rate in terms of body weight of BPH on CR2711–76 showed a strong antibiosis mechanism of resistance, followed by Rathu Heenati and PTB-33. Hence, based on these results, it can be concluded that, CR2711–76 possesses a strong antibiosis mechanism on feeding rate of BPH resistance against Laguna populations when compared with PTB-33, which is the Indian national resistant check for biotype 4. It is noteworthy to mention that, honeydew secretion was completely absent in both CR2711–76 and F_1_ derived from the cross of Jaya × CR2711–76, suggesting the complete dominance reaction of resistance gene *BPH31* to BPH for the Nueva Ecijia BPH populations. Hence, CR2711–76 possessing *BPH31* has a strong antibiosis resistance mechanism. In testing antixenosis, the BPH load decreased after 2 days of infestation and the BPH load was lowest in Rathu Heenati and CR2711–76 on the ninth day after infestation, whereas BPH load was highest in Jaya, followed by TN-1. This result showed that BPH nymphs refused to feed on CR2711–76 and Rathu Heenati and, hence, they moved and settled on susceptible lines (non-preference). Thus, CR2711–76, as a resistance gene donor, possesses an antixenosis mechanism and is not allowing BPH to settle and feed on it unlike Jaya which was highly preferred by BPH insects.

The tolerance mechanism of the resistance results showed the significant difference in the tolerance level among the different BPH resistant lines (CR2711–76, PTB-33 and Rathu Heenati) and cultivar CR2711–76 with the *BPH31* gene has exhibited a high level of tolerance mechanism (Fig. 2d). This study explains that CR2711–76 has a broad-spectrum of resistance with antibiosis, antixenosis, and tolerance mechanisms to different BPH populations of the Philippines and BPH biotype 4 of India. It has been well understood that those varieties or cultivars that possess all three kinds of mechanisms of resistance often show broad-spectrum and stable resistance for a long period of time (Panda and Heinrichs 1983). If the antibiosis and antixenosis mechanisms of resistance genes are overcome by the insect, the tolerance mechanism can work as a second line of defense that may continue to function (Mackenzie 1980). In support, the bioassay results from 2009 to 2014 in India and 2013–2015 in the Philippines (~7 years) proved that, CR2711–76 is stable resistant cultivar (Additional file 9: Figure S9 and Table 1). Hence, CR2711–76 can be considered as a new donor for broad-spectrum resistance to different BPH populations of the Philippines such as Laguna, Isabela, Bicol, and Neuva Ecijia which belongs to three different BPH biotypes, biotype 1, 2 and 3 (Saxena and Barrion 1985) and Indian biotype 4. Similar to this study, IR64 also has all three mechanisms of resistance (antibiosis, antixenosis, and tolerance) and sustained moderate resistance for quite a long period of time (Cohen et al. 1997).

Thirty BPH resistance genes have been reported to date (Deen et al. 2017). Many of these BPH resistance genes identified showed resistance against biotype 1 and 2 are not effective against biotype 4 (Deen et al. 2010). Advances in molecular biology and the availability of recent new molecular genomics approaches have paved the way to the identification and fine mapping of many BPH resistance genes going from *BPH18* in 2006 to *BPH32(t)* in 2016 (Cha et al. 2008; Sun et al. 2006; Qiu et al. 2010; Zhao et al. 2016; Qiu et al. 2012; Du et al. 2009; Lv et al. 2014; Jena et al. 2006; Chen et al. 2006; Myint et al. 2012; Tamura et al. 2014; Huang et al. 2013; Wu et al. 2014; Wang et al. 2015; Ren et al. 2016). For the resistance gene *BPH31*, the initial genetic locus size was around 16 Mb and, hence, the chance of recombination between the genetic locus and the markers was high. We applied a fine-mapping approach to narrow down and reduce the gene-marker recombination. Based on the information available on rice genome sequences and putative functions involved in BPH resistance or biotic stress stimulus in 3000 genome sequences at IRRI, in the OryzaSNP database, and in Nipponbare genomic sequences available in the MSU database by the prediction method, we designed putative linked markers for *BPH31* (Additional file 11: Table S1). Of the 27 InDel markers designed at regular intervals, four were polymorphic and these markers were used for linkage analysis using 151 F_2:3_ mapping populations similar to the earlier approaches (Jena et al. 2006; He et al. 2013; Wu et al. 2014). Linkage analysis was further carried out to find closely linked DNA markers for *BPH31.* Finally, the *BPH31* gene was located in between the markers PA26 and RM2334, which have 79.8% phenotypic variance at LOD 47.64, and the size of the locus was 475 kb. For the mapping experiments, marker and phenotype data were analyzed using QTL IciMapping software ver. 4.0 (Meng et al. 2015) and confirmed with WinQTL Cartographer ver 2.5 (Wang 2012). The derived results and gene location were similar in all the analyzed tools, indicating the high reliability of the results and experiments conducted. To our knowledge, no BPH resistance gene has been reported in the chromosomal region of the *BPH31* locus. However, *BPH13* gene was localized on the same chromosome 3 and, hence, we evaluated their resistance reaction in comparison with that of *BPH31.* This gene was significantly differing in its resistance reaction and the mechanism of resistance as shown in Additional file 1: Figure S1a and b. The genomic fragment of the *BPH31* locus contains minimum of 42 putative genes, of which three candidate genes (*LOC_Os03g46440*, *LOC_Os03g46454,* and *LOC_Os03g46550)* are found to be near the closely linked marker PA26. Gene expression of these genes was studied using online data bases including RiceXPro. This data base retrives transcriptome analysis of different candidate genes (*LOC_Os03g46440*, *LOC_Os03g46454,* and *LOC_Os03g46550*) which were already deposited (Sato et al. 2013). the result from this analysis showed that these genes were involved in biotic stimulus functions. Especially, the gene *LOC_Os03g46550* encodes the LRR domain, which was reported to be involved in BPH resistance for four of the seven BPH resistance genes including *BPH18* (Du et al. 2009; Tamura et al. 2014; Liu et al. 2015; Ji et al. 2016) and close to the flanking marker which showed high co-segregation. This gene found expressing at the early seedling stage like other BPH resistance genes. Similarly, the *BPH31* locus may also lie in the cluster of three candidate genes conferring BPH resistance. Alternatively, one of the three genes may act independently for BPH resistance. However, this needs further study for confirmation (Additional file 13: Table S3).

Jaya is one of the commercially cultivated rice varieties in Southern India but it is highly susceptible to BPH. We selected Jaya for improving BPH resistance through transfer of the new CR2711–76 **-** derived *BPH31* gene in a resistance breeding program using the tightly linked markers PA26 and RM2334. Therefore, the present study focused on improving Jaya by introgressing *BPH31* through marker-assisted backcrossing. Agronomic traits of the improved lines were taken care of as Jaya was preferred by farmers and consumers for its grain quality. The results on agronomic performance showed that introgressed lines were superior for panicle length and grain yield per plant to Jaya while grain characteristics similar to Jaya.

## Conclusion

The present study identified a new, broad-spectrum BPH resistance gene, *BPH31* that was located on the long arm of chromosome 3 by linkage analysis and fine-mapping strategies. This gene not only showed a consistent resistance reaction to Indian BPH biotype 4 but also resistance to several BPH populations collected in The Philippines. This study also revealed that the *BPH31* gene had all three BPH resistance mechanisms responsible for broad-spectrum resistance. The applicability of the gene and the linked markers (PA26 and RM2334) showed promise through introgression lines obtained in the indica rice cultivar Jaya background through MAS. We believe that the improved variety Jaya will facilitate managing BPH problems in Southern India. The *BPH31* gene and the tightly linked InDel type markers can be used in any other BPH resistance breeding programs toward developing rice cultivars with broad-spectrum BPH resistance on the Indian subcontinent.

## Methods

### Plant materials

The variety CR2711–76 developed by NRRI, Cuttack, India, was used as a donor material for the identification of a new BPH resistance gene. A BPH-susceptible variety, Jaya, was used as the recipient parent. A set of 13 BPH NILs, (NIL-*BPH2*, NIL-*BPH3*, NIL-*BPH4*, NIL-*QBPH4 + QBPH6*, NIL-*BPH9*, NIL-*BPH10*, NIL-*BPH17*, NIL-*BPH18*, NIL-*BPH20*, NIL-*BPH21*, NIL-*BPH25*, NIL-*BPH26,* and NIL-*BPH25 + BPH26*) and the *BPH13* donor line were developed and maintained in the Novel Gene Resources (NGR) laboratory, Plant Breeding Division of IRRI (Jena et al. unpublished data). These NILs were used to comparatively assess the resistance/susceptible reactions of the new resistance gene with already reported genes and to assess the differences in mechanism of resistance. PTB-33 and Rathu Heenati were used as BPH-resistant checks while Taichung Native-1 (TN-1) and Jaya were used as susceptible check cultivars. The detected gene in the present study was designated as *BPH31* because this gene was detected during 2015. At that time only 30 BPH reistance genes were identified and designated. Since this was the new gene, it was designated as *BPH31* after *BPH30* which was later considered by Ren et al. 2016 and hence, they named their gene as *BPH32*.

### Development of mapping populations

A cross between the resistant variety CR2711–76 and Jaya was made and F_1_ plants were produced. The true F_1_ plants were selfed and they produced F_2_ seeds. In addition, the F_1_ plants were further backcrossed to the recurrent parent Jaya to generate BC_1_F_1_ seeds. A total of 151 F_2_ and BC_1_F_1_ plants were genotyped and were selfed to generate F_3_ and BC_1_F_2_ progenies, respectively. The F_3_ plants were phenotyped for BPH resistance or susceptibility. Foreground selection was practiced to generate BC_2_F_1_ progenies after backcrossing with Jaya as the recurrent parent, based on the identified gene flanking marker genotype data. Phenotypic selection and background analysis were performed to generate BC_3_F_1_ plants {introgressed lines (ILs)} that possessed the new BPH resistance gene. To confirm their resistance and to validate the co-segregation of the newly developed markers, BC_2_F_1_ plants were selfed and the BC_2_F_2_ plants were used for marker validation (Additional file 7: Figure S7).

### BPH population and bioassay for resistance

A pure BPH population (biotype 4) was developed from a single colony of BPH and was grown on the susceptible variety Jaya in a cage in Entomology Division of the Zonal Agricultural Research Station (ZARS), V.C. Farm, Mandya, Karnataka, India. The susceptible check variety TN-1 was used for feeding different BPH populations of the Philippines (Isabela, Nueva Ecija, Laguna and Bicol). Four BPH populations are the representative populations of the Philippines in terms of geographical locations: the Isabela, Nueva Ecijia, Laguna and Bicol provinces belongs to Region II, Region III, Region IV and Region V. These populations are the representative BPH populations of biotype 1, 2 and 3 (Saxena and Barrion 1985). The bioassay was done following the method of Jena et al. (2006) with minor modifications. Seedlings at the two- to three-leaf stage were infested with second- or third-instar nymphs at a density of 10–12 nymphs per seedling. Once the standard susceptible check varieties Jaya and TN-1 started wilting (90%), data were recorded as seedling survival rate and finally expressed as a standard evaluation system (SES) score (IRRI, 1996). Higher BPH scores indicate susceptibility and the lowest score indicates an immune response of the genotype. For the evaluation of parental lines, a minimum of 25 seedlings were maintained per test entry with three replications. For phenotyping of the mapping population, seedlings of F_3_ families of the F_2_ mapping population (Jaya × CR2711–76) were planted into randomly selected rows in the glasshouse. Evaluations were based on the degree of susceptibility of the S check. Genotypes with a BPH response (RR: homozygous resistant, RS: segregating heterozygous, SS: homozygous susceptible) of the 151 F_2_ individuals were determined by assaying the phenotypes (R or S) of the corresponding F_3_ progenies. A complete resistance score (0–1) was given to progenies expressing 91–100% seedling survival, resistance scores of 2–3 were for progenies with 76–90% seedling survival, scores of 4–7 were for progenies with 11–75% survival, and scores of 8–9 were for progenies with complete susceptibility and 0–10% survival.

### Mechanisms of BPH resistance

The present study explored all three kinds of resistance mechanisms: antibiosis, antixenosis, and tolerance.

#### Antibiosis

The antibiosis mechanism of resistance was measured based on two parameters: **a**ntibiosis on nymph survival and antibiosis on feeding rate.

#### Antibiosis on nymph survival

In this method of determining antibiosis mechanism, about 20 pre-germinated seedlings of each cultivar were grown in a 10 cm diameter clay pot. The pots were randomly placed in a 68 cm × 42 cm × 16 cm plastic seed-box with about 2 cm of water maintained in the bottom until completion of the experiment. Seedlings were thinned to 6 per pot 7 days after seeding, and at the third-leaf stage were infested with 2nd -instar BPH nymphs at the rate of 10 insects per plant. The potted plants were then covered with cylindrical Mylar cages (13 × 50 cm). The nymphs per plant were counted 4-day post-infestation. The experiment was repeated twice; 6 individual plants were measured for each cultivar per replicate. Number of nymphs on each plant was finally expressed as nymph survival rate and the genotype which has highest nymph survival rate declared as susceptible after comparing with susceptible and resistant check and vice versa (Heinrichs et al. 1985; Hu et al. 2012).

#### Antibiosis on feeding rate

This is another method of determining the level of antibiosis based on the area of honey dew secreted and weight of the BPH insects. The experiment was conducted by following the methodology prescribed by Heinrichs et al. 1985 with slight modifications. Exactly 10 female hoppers (adult gravids) were introduced after starving for 1–1.5 h into each cage through the top of the inverted plastic cup where the stem of the plant passes through. The opening of the cup is plugged with cotton to prevent escape of the insects. Spots on the filter paper are evident as soon as honeydew is excreted. After the hoppers have fed for 24 h, filter papers were removed from the cage. Collected filter paper is placed on A4 size white paper as 2 or 4 filter papers per white paper. A 1 cm line was drawn on the A4 size white paper at the top of the filter paper this will be used to set scale for measuring the area of spot by using ImageJ software. BPH adults were collected and oven dried for 24 h. After 24 h oven dried BPH adult females were weighed in 0.001 mg sensitive weighing balance for estimating body weight.

#### Antixenosis

##### Antixenosis on nymphs on seedlings

Another mechanism of resistance, antixenosis, was assayed based on the preference of test lines by the BPH for feeding. This study on the level of antixenosis was conducted simultaneously with the conventional seed box screening test (Heinrichs et al. 1985). The first scoring was recorded after 24 h of infestation of BPH nymphs at two to three-leaf stages of plants and next scoring was done after every 24 h for number of BPH nymphs per hill until 90% of TN-1 starts wilting. Genotypes which were most preferred by BPH were declared as susceptible and do not possess antixenosis mechanism and vice versa*,* after comparing with the load of BPH nymphs on susceptible and resistant checks following the method of Heinrichs et al. (1985). Each genotype was replicated three times and the whole experiment was repeated twice to attain more accurate results and appropriate conclusion.

#### Tolerance

Experiments on testing the tolerance mechanism were also carried out by measuring parameters such as functional plant loss index (FPLI) and tolerance index (TI) by following the methodology described by Panda and Heinrichs (1983) and Hu et al. (2015) with a few modifications.

About 20 pre-germinated seedlings of each variety were sown in a 10 cm-diameter clay pot placed in a 68 cm × 42 cm × 16 cm plastic seed box. About 2 cm of water was maintained in the seed-box until the experiment was finished. Seven days after sowing, seedlings were thinned to 6 plants per pot. At the third-leaf stage, 1st instar nymphs (10 numbers) were placed on each plant. The potted plants were then covered with cylindrical Mylar cages (13 cm × 50 cm). Three control plants without insects were maintained for each line. When the control TN1 plants started to wilt, the experiment was stopped and the BPH nymphs were collected from each line, oven-dried for 48 h and weighed. Infested and non-infested plants were removed from the pots along with the roots, washed thoroughly, air dried for 3 h, then dried in an oven at 65 °C for 60 h and weighed. The level of tolerance was assessed using the parameters FPLI and TIs. A scatter plot was set up with the x-axis representing the weight of insects and the y-axis representing the FPLI. The transept of average BPH weight and the regression line divided the scatter plot into four sectors representing high antibiosis and low tolerance, high tolerance and low antibiosis, high antibiosis and high tolerance, and low antibiosis and low tolerance (susceptibility), as described by Panda and Heinrichs (1983). Six replicates of each cultivar and four replications of whole experiment were carried out. The FPLI and TI were estimated by following formula,

$$ FPLI=100-\left(\frac{dry\  weight of infested plant}{dry\  weight of non- infested plant}\right)\ x\ \left(1-\frac{damage}{9}\right)x\ 100 $$

$$ TI=\frac{BPH\ w eight\  on\  test line}{BPH\  weight\  on\  susceptible control\ \left( TN-1\right)} $$

### Assessment of broad-spectrum antibiosis of CR2711–76 against different populations of BPH

Antibiosis experiments were also conducted to assess the broad spectrum resistance of resistant donor CR2711–76 using different populations of BPH, Laguna, Isabela, Bicol and Nueva Ecijia of the Philippines; and biotype 4 of India with three replications. The varieties which show resistance reaction to all the different BPH populations of the Philippines and the biotype 4 of India were declared as genotype/s with broad-sense resistance.

### Inheritance of antibiosis on feeding rate

The inheritance pattern of antibiosis on feeding rate between Jaya and CR2711–76 was studied, The F_1_s generated from the cross of Jaya and CR2711–76, parental lines along with the checks were bioassayed for antibiosis on feeding rate for Laguna and Nueva Ecijia BPH colonies in two replications.

### Fine mapping of the *BPH31* gene

Genomic DNA was extracted by the modified CTAB method as described by Kim et al. (2011). Simple sequence repeat (SSR) and sequence tagged site (STS) markers were used to identify the gene and its introgression. PCR reactions were carried out with standard PCR buffers and conditions. The thermal profile followed was 4 min at 94 °C, followed by 35 cycles of 1 min at 94 °C, 30 s at 55 °C, and 1 min at 72 °C, with a final extension of 10 min at 72 °C. Amplification products were separated by electrophoresis in 3.5% agarose gel or 8.0% non-denaturing PAGE. In total, 812 SSR and STS primer sets were tested for the polymorphism on the parental lines, Jaya and CR2711–76. Among them, 107 markers showed polymorphic PCR bands between the parents and they were potentially used for characterizing the alleles existing among the 151 F_2_ plants. MAPMAKER/EXP 3.0 (Lincoln et al. 1992) was used to construct the linkage map from the 107 markers spanning the 12 rice chromosomes. The Kosambi mapping function (Kosambi 1944) was used to convert recombination fractions to map distances in centiMorgans (cM).

The precisely estimated phenotypic data and well-distributed marker genotype data were used for locating genomic regions conferring BPH resistance. QTL IciMapping software ver. 4.0 (Meng et al. 2015) with 1000 permutations at 0.01 significance LOD threshold and its results were validated with WinQTL cartographer ver. 2.5 (Wang 2012) considering the same threshold parameters.

Based on the whole genome sequence of reference genome Nipponbare*,* TIGR prediction method (http://rice.plantbiology.msu.edu/), available information from the 3000 Rice Genome Project (3 K RGP), and OryzaSNP (http://snp-seek.irri.org/) database, 27 putative InDel markers were designed to narrow down the *BPH31* locus and were surveyed for polymorphism between Jaya and CR2711–76. The markers detecting polymorphism between Jaya and CR2711–76 were further tested on the F_2_ mapping population and these markers segregated for marker alleles in the F_2_ progenies. Furthermore, QTL analysis was performed again by retaining the same threshold parameters to identify the markers closely linked to *BPH31*. All candidate genes within the identified region were analyzed based on the information available in NCBI (https://www.ncbi.nlm.nih.gov/unigene), TIGR release 7.1 (http://rice.plantbiology.msu.edu/), RiceXPro (Sato et al. 2013; http://ricexpro.dna.affrc.go.jp/) and Rice oligonucleotide array data base (http://www.ricearray.org/expression/meta_analysis.shtml).

### Validation of *BPH31* flanking markers for MAS

To investigate the accuracy of the identified marker, a BC_2_F_2_ population consisting of 268 individuals derived from a heterozygous line of BC_2_F_1_, BC_1_F_1_–61-BC_2_F_1_–19-13-(33–13)-BC_2_F_2_–11–20, and a homozygous recessive line, BC_1_F_1_–35-BC_2_F_1_–8-12(17–14)-BC_2_F_2_–271–280, were screened for BPH resistance using the modified standard seed box method (Jena et al. 2006). For this experiment, 149 BC_2_F_2_ seeds obtained from a heterozygous line and 119 BC_2_F_2_ seeds from a homozygous recessive line were sown in blue trays. The segregation for BPH resistance was studied in the populations by directly assaying the phenotype of the BC_2_F_2_ individuals. The genotype and phenotype of all these BC_2_F_2_ individuals were compared and percent co-segregation for markers within the preliminary mapped locus was computed. The markers with high co-segregation were identified and recommended for marker assisted introgression.

### Background genotyping and construction of a graphical genotype map

To determine the genetic background recovery of the BC_2_F_1_ progenies, a high-density SNP marker genotyping system, Infinium 6 K SNP chip assay was employed. The Infinium platform detects SNP alleles by adding a fluorescence-labeled allele-specific nucleotide (allele-specific hybridization) via single-base extension and subsequent detection of the fluorescence color. Generated HapMap format SNPs were sorted and filtered out for no calls and monomorphism between the parents before they were used for the computation of percent recovery of the recurrent parent genome. Finally, the graphical genotype map was drawn based on the filtered SNP genotype data using the web tool PhenoGram (http://visualization.ritchielab.psu.edu/phenograms/plot).

### Statistical analysis

A randomized complete block design (RCBD) was employed to analyze the data obtained from the bioassay of parental lines to test the significance of the experiments. Fisher’s least significant difference (LSD) test and Duncan’s multiple range test (DMRT) at α = 0.05 and/or 0.01 significance were used to compare the means of the test entries and to infer the significant difference between the cultivars under study. Maximum likelihood (multiple regression analysis) was employed for composite interval mapping for preliminary and fine mapping of *BPH31.* Chi-square goodness-of-fit was employed to fit the segregation pattern of *BPH31* based on the genotypic and phenotypic ratios, to study the genetics of resistance and also inheritance of the antibiosis mechanism. The Chi-square test for goodness-of-fit was performed manually using MS-Excel. Simple linear regression and pooled regression analysis were calculated in the tolerance experiment to classify the different mechanisms of resistance into different sectors (Panda and Heinrichs 1983).

### Statement

Statement I confirm that we have followed the guide lines of the government of The Philippines and the policies of IRRI for growing rice plants and carrying out research study.

## Addtional files

Additional file 1: Figure S1. Graphical representation of area of honeydew secretion of **(a)** first experiment, **(b)** repeated experiment for Isabela BPH colony, (**c)** for Bicol BPH colony, and (**d)** for Nueva Ecijia BPH colony; among the test genotypes including parental lines, Jaya and CR2711–76; P_1_, P_2_, 13 t_,_ RH, and F_1_ are Jaya, CR2711–76, *BPH13* donor line, Rathu Heenati, and F_1_ is the hybrid of Jaya × CR2711–76, respectively; RH and TN-1 are resistant and susceptible checks. (PPTX 178 kb)
Additional file 2: Figure S2. Photograph depecting the area of honeydew secreated when Lagun colony of BPH fed on TN-1, Rathu Heenati, CR2711–76 and Jaya testlines. (PPTX 287 kb)
Additional file 3: Figure S3. Rice molecular linkage map obtained from 107 SSR and STS markers of 151 F_2:3_ mapping population of the cross combination Jaya × CR2711–76. Chromosome numbers are presented on the top of each linkage group, marker names are presented on the right side of each linkage group, and their intervals in cM are presented on the left side of the linkage group. This linkage group was generated by QTL IciMapping software considering the marker position and order retrieved from MAPMAKER ver. 2.0. (PPTX 160 kb)
Additional file 4: Figure S4. The schematic diagram of primary mapped BPH resistance locus *BPH31* on chromosome 3 flanked by RM251 and RM2334 with 48.40 LOD and 80.71% phenotypic variance explained derived from 151 F_2:3_ mapping population in the whole-genome view. This result was retrieved from the linkage analysis of (**a)** QTL IciMapping ver. 4.0 (Meng et al. 2015) and (**b)** WinQTL cartographer ver. 2.5 (Wang 2012) software package. (PPTX 658 kb)
Additional file 5: Figure S5. (**a)** Raw signal intensity bar graph (in silico gene expression analysis as available in public domain, http://ricexpro.dna.affrc.go.jp/GGEP/graph-view.php?featurenum=29277) showing the expression intensity of *LOC_Os03g46550* gene*.* (**b**) Preferential gene expression at different developmental stages of the rice plant. (**c**) Tissue-specific differential expression of *LOC_Os03g46550* (based on information available at http://www.ricearray.org/expression/meta_analysis.shtml). (PPTX 706 kb)
Additional file 6: Figure S6. Photograph showing the bioassay result of negative and positive *BPH31* BC_2_F_2_s of marker validation experiment at maximum lethal stage (ninth day after infestation) for the Laguna BPH population. (PPTX 296 kb)
Additional file 7: Figure S7. Schematic diagram showing material generation for the identification and introgression of *BPH31*. (PPTX 71 kb) 
Additional file 8: Figure S8. Photograph showing the typical bioassay conducted using CR2711–76, Jaya, Rathu Heenati and TN-1 along with other test lines. (PPTX 542 KB)
Additional file 9: Figure S9. Graphical representation of 7 years BPH (biotype 4) bioassay result of parental lines and checks. (PPTX 171 KB)
Additional file 10: Figure S10. Graphical representation of comparative resistance reaction of BPH31 with already identified genes against Laguna BPH colony. (PPTX 128 kb)
Additional file 11: Table S1. List of newly designed InDel markers for fine mapping of *BPH31*. (PPTX 77 kb)
Additional file 12: Table S2. List of candidate genes identified within the fine-mapped region (475 kb) along with their putative function. (PPTX 86 kb)
Additional file 10: Table S3. Seven years BPH (biotype 4) bioassay result of parental lines and checks. Phenotypic values are the average seedling survival rates. (PPTX 55 kb)

## Abbreviations

BPH: Brown planthopper
CTAB: Cetyl trimethylammonium bromide
DMRT: Duncan’s multiple range test
FPLI: Functional plant loss index
LOD: Logarithms of odds
LSD: Least significant difference
MAS: Marker assisted selection
NCBI: National centre for biotechnology information
NIL: Near isogenic line
PCR: Polymerase chain reaction
QTL: Qunatitative trait locus
RCBD: Randomized complete block design
SES: Standard evealuation system
SNP: Single nucleotide polymorphism
SSR: Simple sequence repeat
STS: Sequence tagged site
TI: Tolerance index
TN-1: Tai-chung Native-1
ZARS: Zonal agricultural research station
