# Supplementary material for: Identification and fine mapping of a new gene, BPH31 conferring resistance to brown planthopper biotype 4 of India to improve rice, Oryza sativa L
Source: Rice (N Y). 2017 Aug 31;10:41. doi: 10.1186/s12284-017-0178-x (PMC5578944; doi:10.1186/s12284-017-0178-x)
Supplement: Additional file 1: Figure S1. — Graphical representation of area of honeydew secretion of (a) first experiment, (b) repeated experiment for Isabela BPH colony, (c) for Bicol BPH colony, and (d) for Nueva Ecijia BPH colony; among the test genotypes including parental lines, Jaya and CR2711–76; P1, P2, 13 t, RH, and F1 are Jaya, CR2711–76, BPH13 donor line, Rathu Heenati, and F1 is the hybrid of Jaya × CR2711–76, respectively; RH and TN-1 are resistant and susceptible checks. (PPTX 178 kb) [file 12284_2017_178_MOESM1_ESM.pptx]

## Slide 1
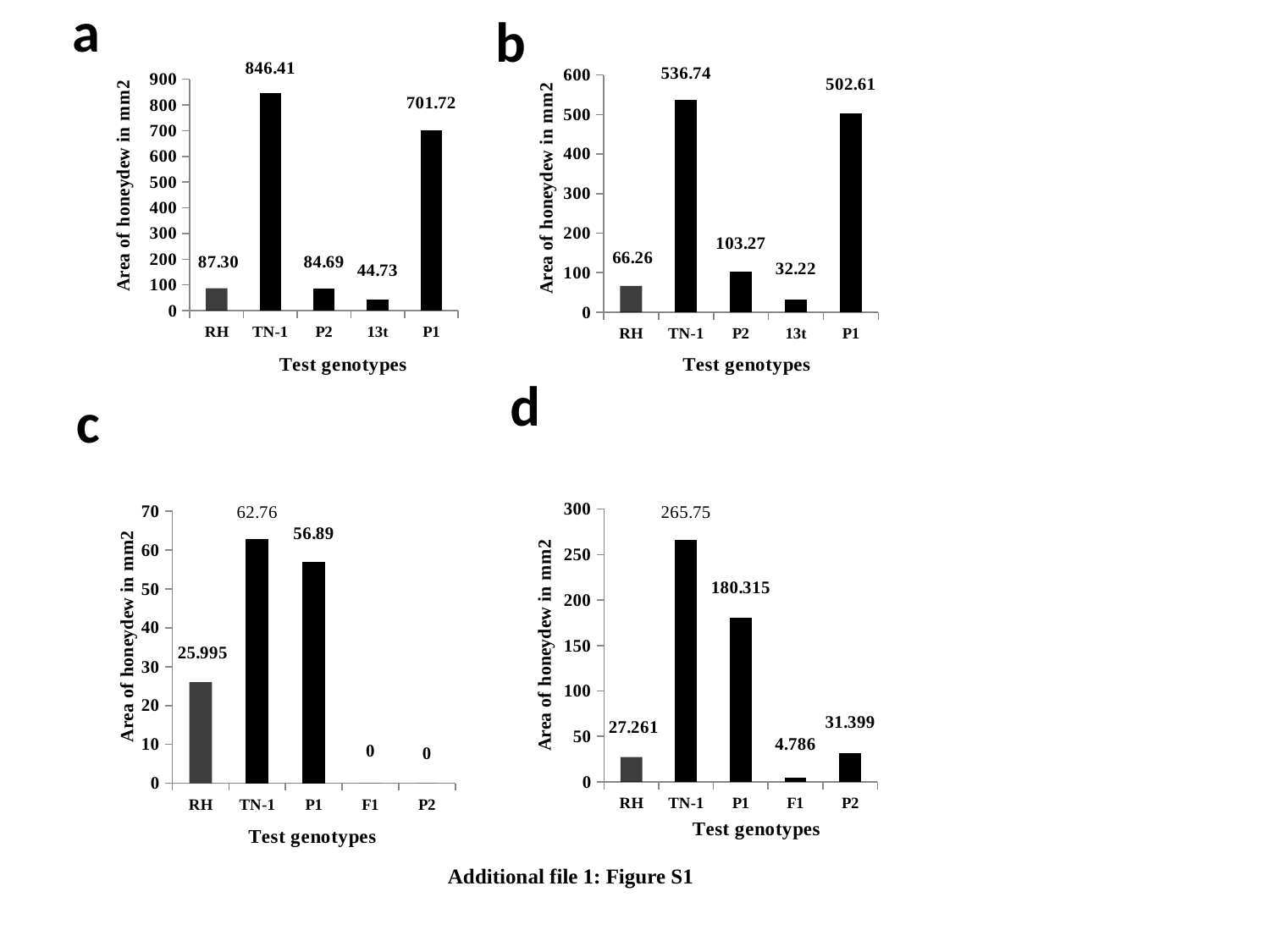

b
### Chart
| Category | RH TN-1 P2 13t P1 |
|---|---|
| RH | 66.2565 |
| TN-1 | 536.7380000000005 |
| P2 | 103.273 |
| 13t | 32.21900000000001 |
| P1 | 502.605 |a
### Chart
| Category | RH TN-1 P2 13t P1 |
|---|---|
| RH | 87.29650000000002 |
| TN-1 | 846.4119999999995 |
| P2 | 84.68599999999998 |
| 13t | 44.728500000001446 |
| P1 | 701.72 |d
c
### Chart
| Category | RH TN-1 P1 F1 P2 |
|---|---|
| RH | 27.26099999999999 |
| TN-1 | 265.75350000000003 |
| P1 | 180.315 |
| F1 | 4.786 |
| P2 | 31.399 |
### Chart
| Category | RH TN-1 P1 F1 P2 |
|---|---|
| RH | 25.994999999999987 |
| TN-1 | 62.76250000000127 |
| P1 | 56.89 |
| F1 | 0.0 |
| P2 | 0.0 |
Additional file 1: Figure S1
