# Supplementary figures and images for: Identification and fine mapping of a new gene, BPH31 conferring resistance to brown planthopper biotype 4 of India to improve rice, Oryza sativa L
Source: Rice (N Y). 2017 Aug 31;10:41. doi: 10.1186/s12284-017-0178-x (PMC5578944; doi:10.1186/s12284-017-0178-x)

## Slide 1
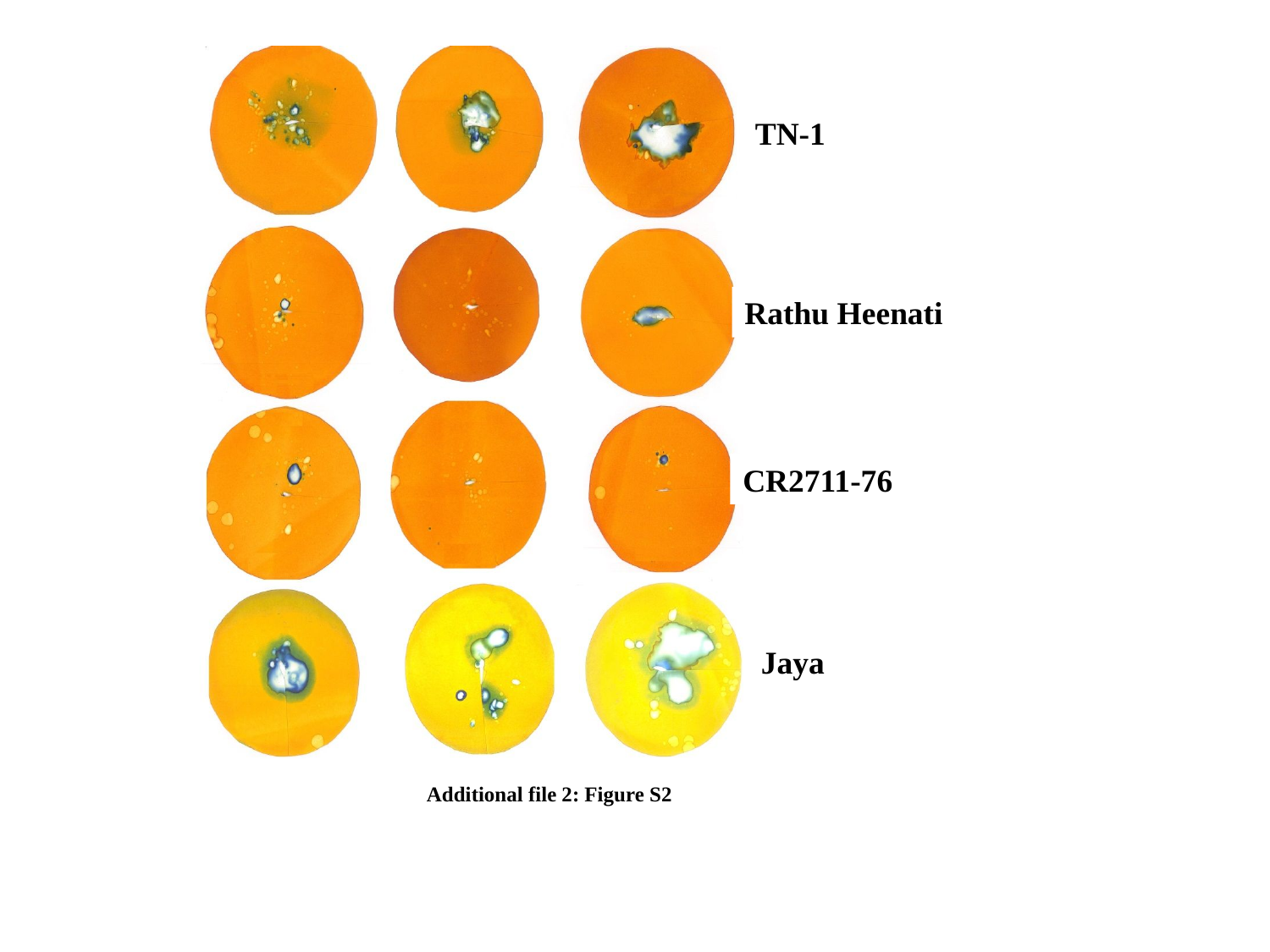

TN-1
Rathu Heenati
CR2711-76
Jaya
Additional file 2: Figure S2

Supplement: Additional file 2: Figure S2. — Photograph depecting the area of honeydew secreated when Lagun colony of BPH fed on TN-1, Rathu Heenati, CR2711–76 and Jaya testlines. (PPTX 287 kb) [file 12284_2017_178_MOESM2_ESM.pptx]

## Slide 1
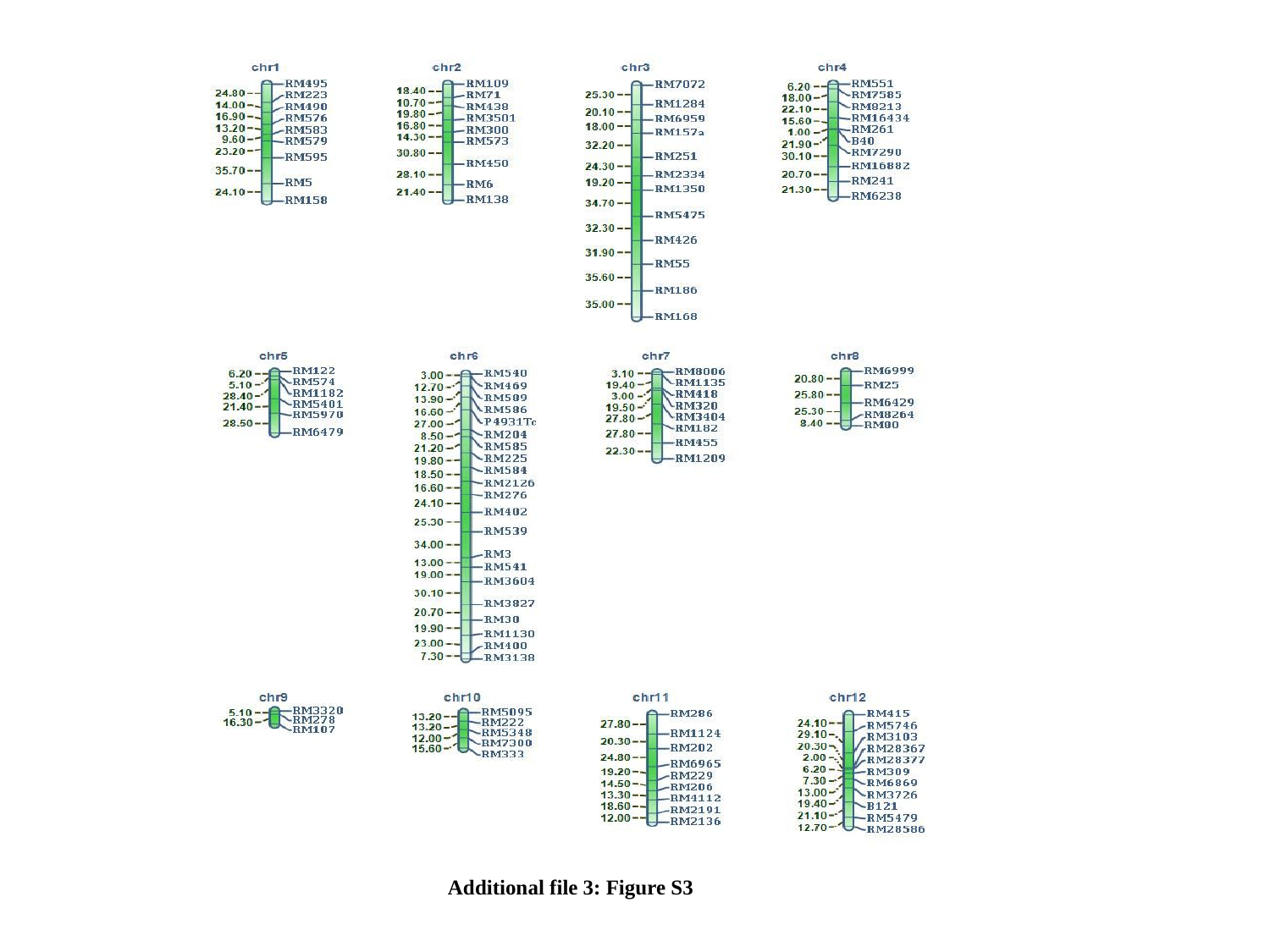

Additional file 3: Figure S3

Supplement: Additional file 3: Figure S3. — Rice molecular linkage map obtained from 107 SSR and STS markers of 151 F2:3 mapping population of the cross combination Jaya × CR2711–76. Chromosome numbers are presented on the top of each linkage group, marker names are presented on the right side of each linkage group, and their intervals in cM are presented on the left side of the linkage group. This linkage group was generated by QTL IciMapping software considering the marker position and order retrieved from MAPMAKER ver. 2.0. (PPTX 160 kb) [file 12284_2017_178_MOESM3_ESM.pptx]

## Slide 1
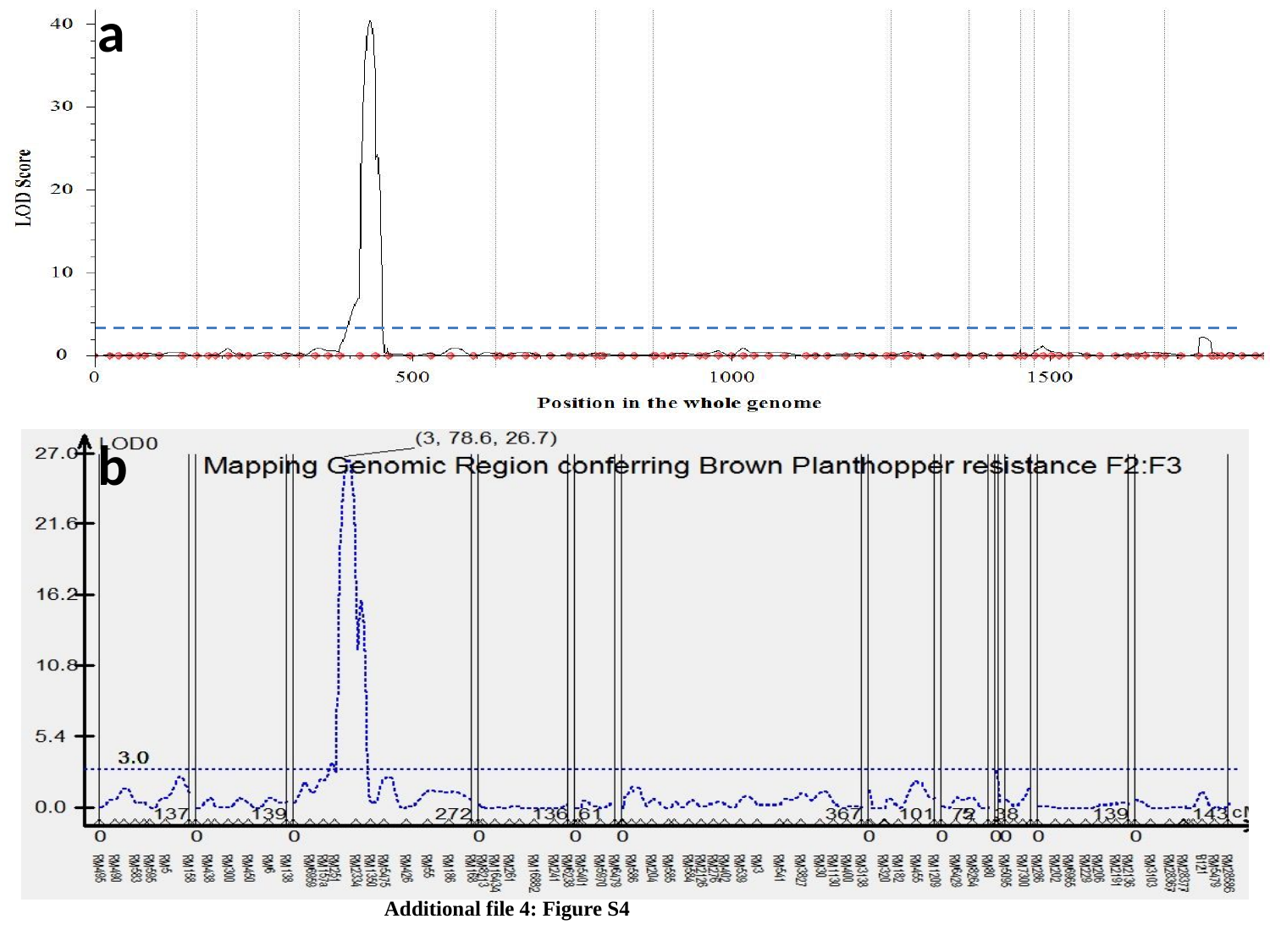

a
b
Additional file 4: Figure S4

Supplement: Additional file 4: Figure S4. — The schematic diagram of primary mapped BPH resistance locus BPH31 on chromosome 3 flanked by RM251 and RM2334 with 48.40 LOD and 80.71% phenotypic variance explained derived from 151 F2:3 mapping population in the whole-genome view. This result was retrieved from the linkage analysis of (a) QTL IciMapping ver. 4.0 (Meng et al. 2015) and (b) WinQTL cartographer ver. 2.5 (Wang 2012) software package. (PPTX 658 kb) [file 12284_2017_178_MOESM4_ESM.pptx]

## Slide 1
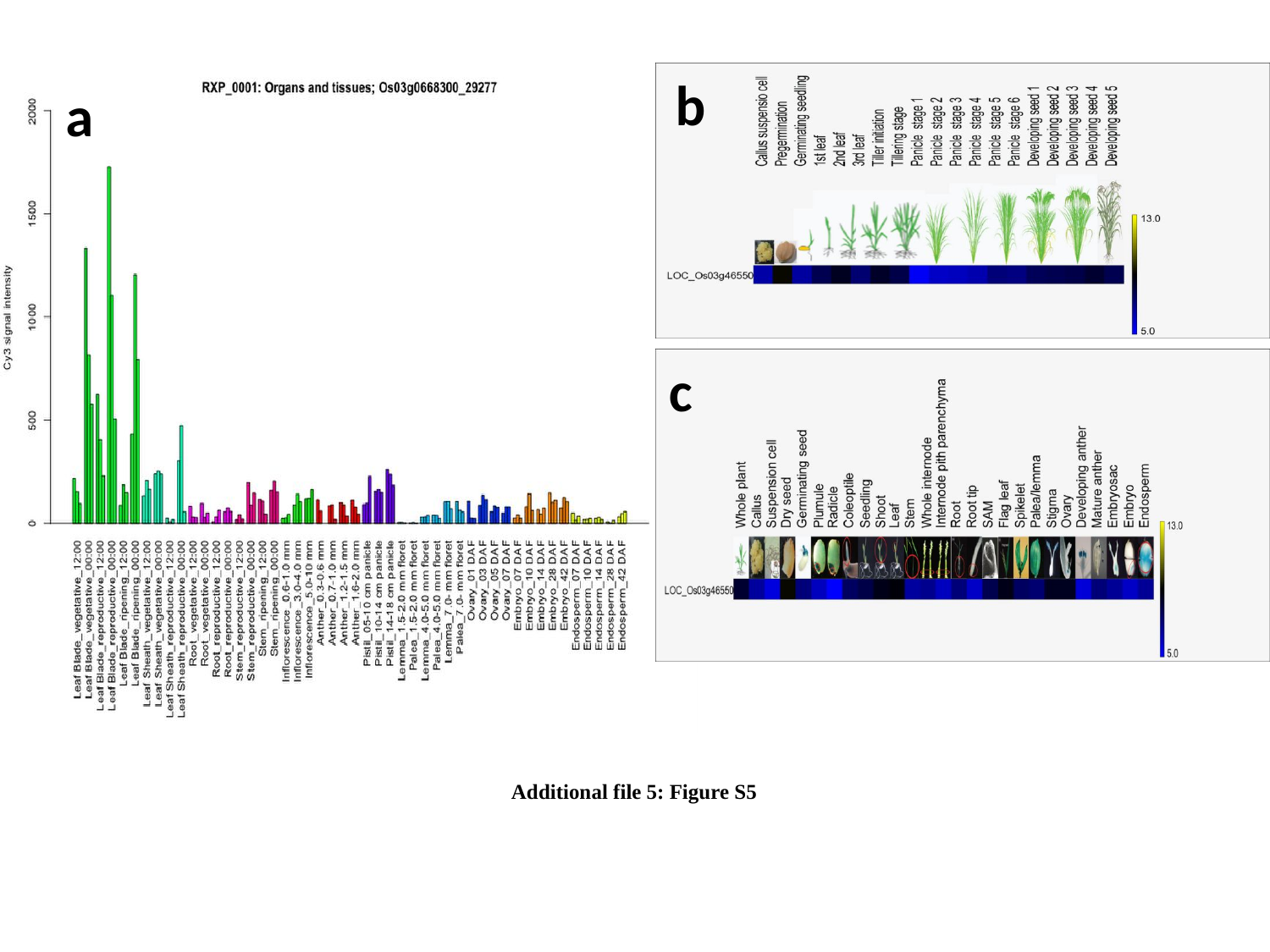

b
a
c
Additional file 5: Figure S5

Supplement: Additional file 5: Figure S5. — (a) Raw signal intensity bar graph (in silico gene expression analysis as available in public domain, http://ricexpro.dna.affrc.go.jp/GGEP/graph-view.php?featurenum=29277) showing the expression intensity of LOC_Os03g46550 gene. (b) Preferential gene expression at different developmental stages of the rice plant. (c) Tissue-specific differential expression of LOC_Os03g46550 (based on information available at http://www.ricearray.org/expression/meta_analysis.shtml). (PPTX 706 kb) [file 12284_2017_178_MOESM5_ESM.pptx]

## Slide 1
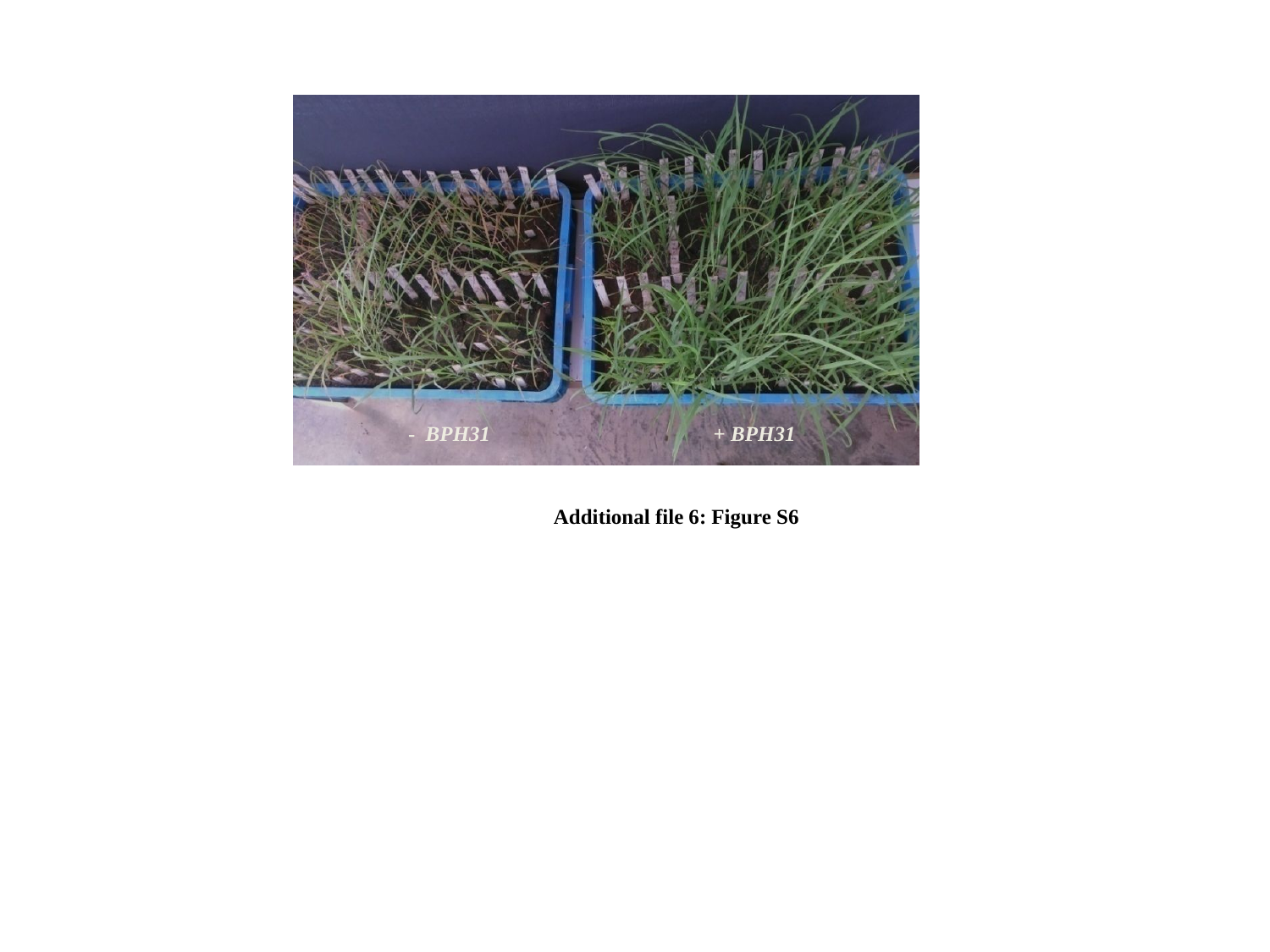

- BPH31
+ BPH31
Additional file 6: Figure S6

Supplement: Additional file 6: Figure S6. — Photograph showing the bioassay result of negative and positive BPH31 BC2F2s of marker validation experiment at maximum lethal stage (ninth day after infestation) for the Laguna BPH population. (PPTX 296 kb) [file 12284_2017_178_MOESM6_ESM.pptx]

## Slide 1
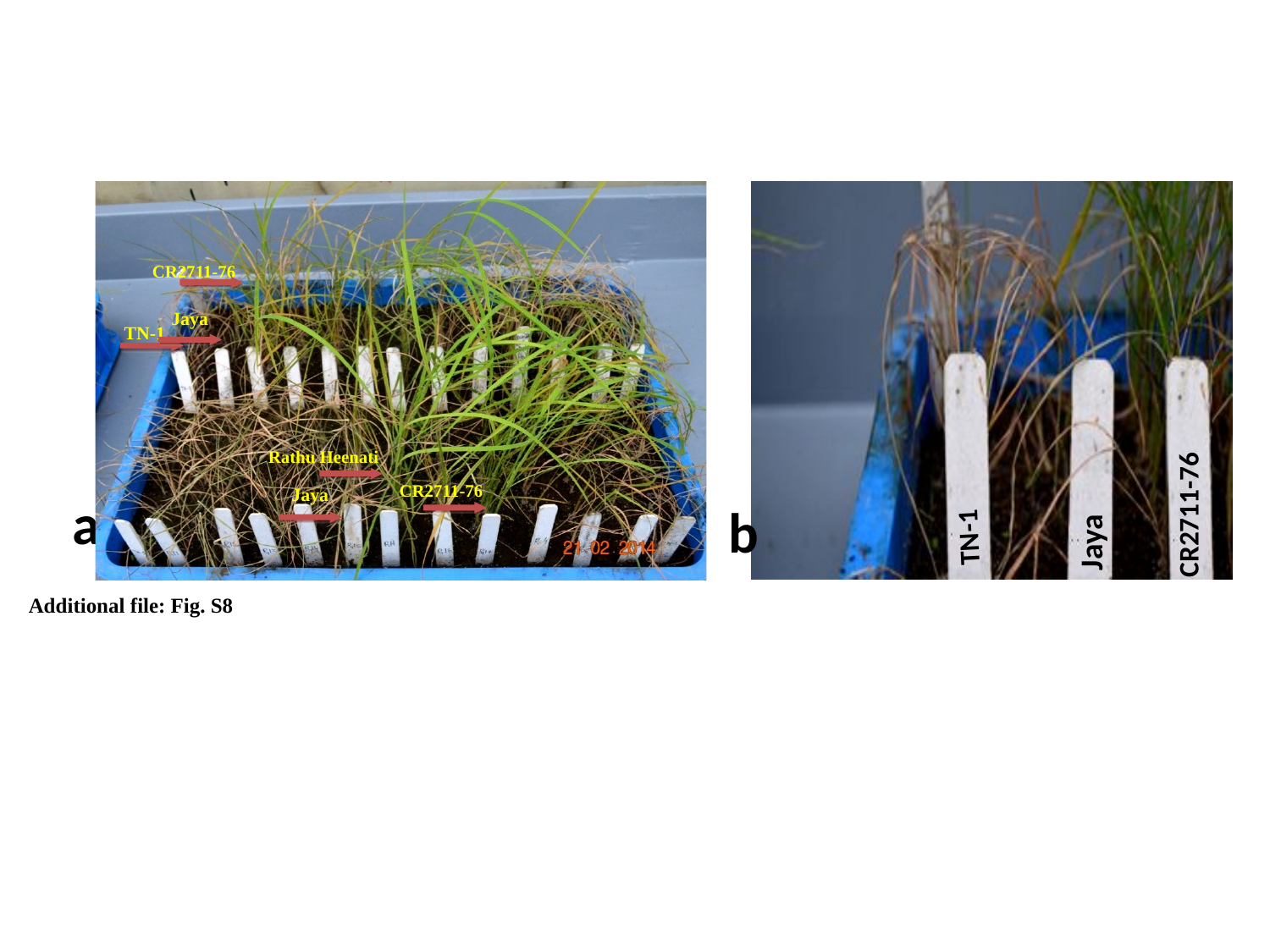

CR2711-76
Rathu Heenati
CR2711-76
TN-1
CR2711-76
TN-1
CR2711-76
Jaya
a
Jaya
b
Additional file: Fig. S8
Jaya
Jaya

Supplement: Additional file 8: Figure S8. — Photograph showing the typical bioassay conducted using CR2711–76, Jaya, Rathu Heenati and TN-1 along with other test lines. (PPTX 542 KB) [file 12284_2017_178_MOESM8_ESM.pptx]

## Slide 1
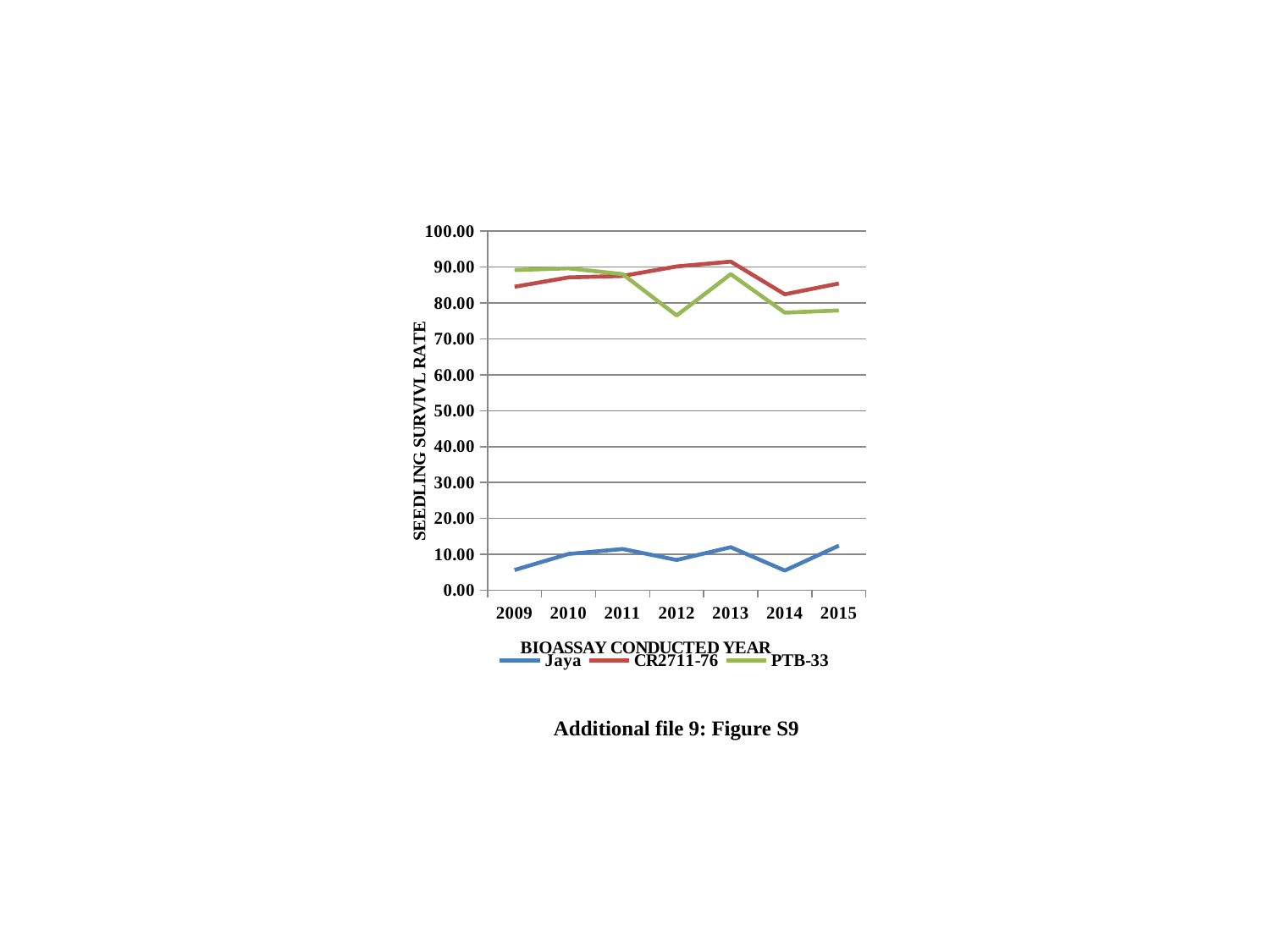

### Chart
| Category | Jaya | CR2711-76 | PTB-33 |
|---|---|---|---|
| 2009 | 5.6499999999999995 | 84.5 | 89.14999999999999 |
| 2010 | 10.120000000000001 | 87.1 | 89.63 |
| 2011 | 11.5 | 87.5 | 88.0 |
| 2012 | 8.450000000000003 | 90.14999999999999 | 76.5 |
| 2013 | 12.0 | 91.5 | 88.0 |
| 2014 | 5.5 | 82.4 | 77.3 |
| 2015 | 12.43 | 85.4 | 77.9 |Additional file 9: Figure S9

Supplement: Additional file 9: Figure S9. — Graphical representation of 7 years BPH (biotype 4) bioassay result of parental lines and checks. (PPTX 171 KB) [file 12284_2017_178_MOESM9_ESM.pptx]
