# Supplementary material for: Identification and fine mapping of a new gene, BPH31 conferring resistance to brown planthopper biotype 4 of India to improve rice, Oryza sativa L
Source: Rice (N Y). 2017 Aug 31;10:41. doi: 10.1186/s12284-017-0178-x (PMC5578944; doi:10.1186/s12284-017-0178-x)
Supplement: Additional file 7: Figure S7. — Schematic diagram showing material generation for the identification and introgression of BPH31. (PPTX 71 kb) [file 12284_2017_178_MOESM7_ESM.pptx]

## Slide 1
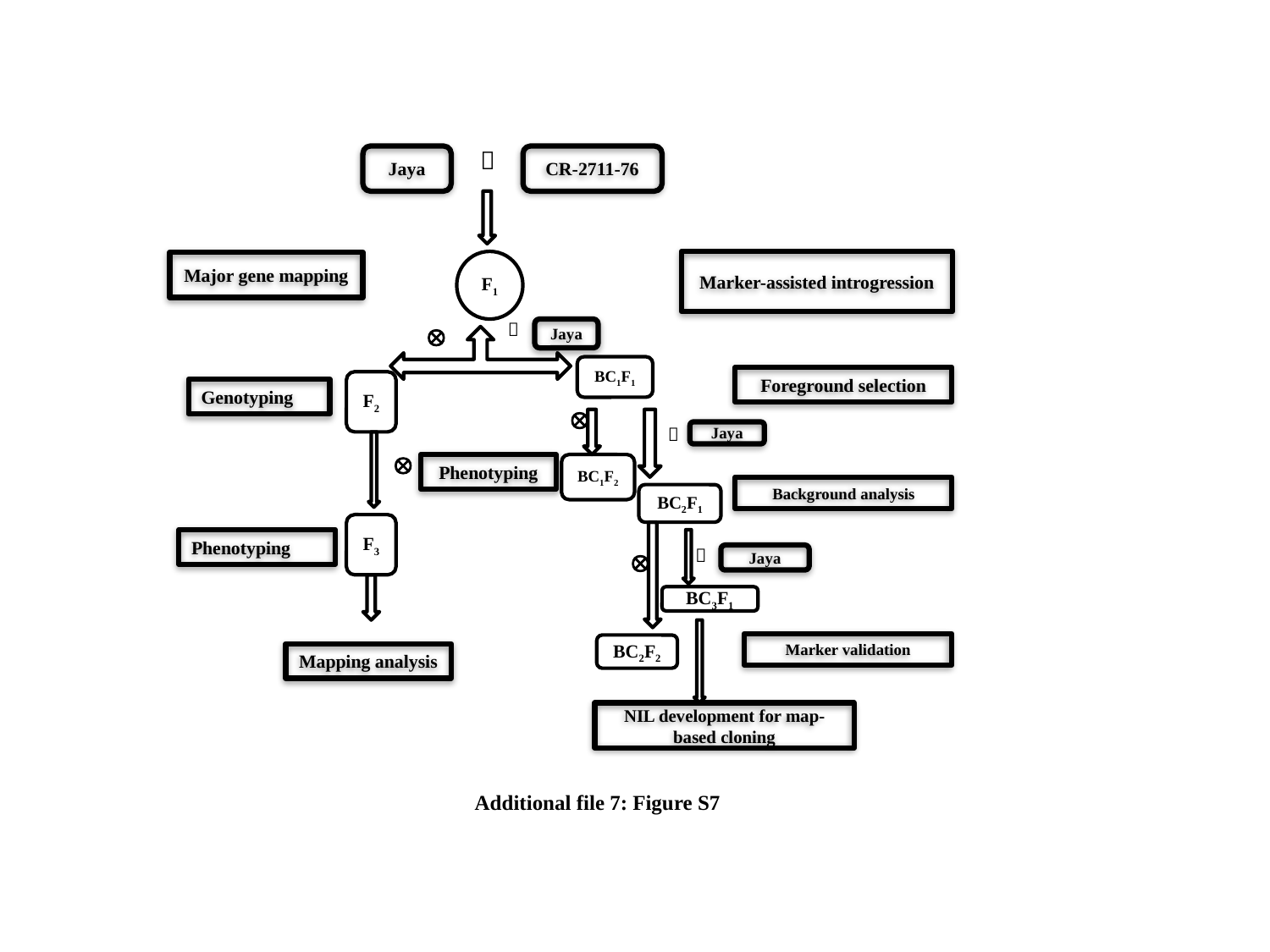


Jaya
CR-2711-76
F1
Marker-assisted introgression
Major gene mapping


Jaya
BC1F1
Foreground selection
F2
Genotyping


Jaya

Phenotyping
BC1F2
Background analysis
BC2F1
F3
Phenotyping


Jaya
BC3F1
Marker validation
BC2F2
Mapping analysis
NIL development for map-based cloning
Additional file 7: Figure S7
