# Supplementary material for: Identification and fine mapping of a new gene, BPH31 conferring resistance to brown planthopper biotype 4 of India to improve rice, Oryza sativa L
Source: Rice (N Y). 2017 Aug 31;10:41. doi: 10.1186/s12284-017-0178-x (PMC5578944; doi:10.1186/s12284-017-0178-x)
Supplement: Additional file 10: Figure S10. — Graphical representation of comparative resistance reaction of BPH31 with already identified genes against Laguna BPH colony. (PPTX 128 kb) [file 12284_2017_178_MOESM10_ESM.pptx]

## Slide 1
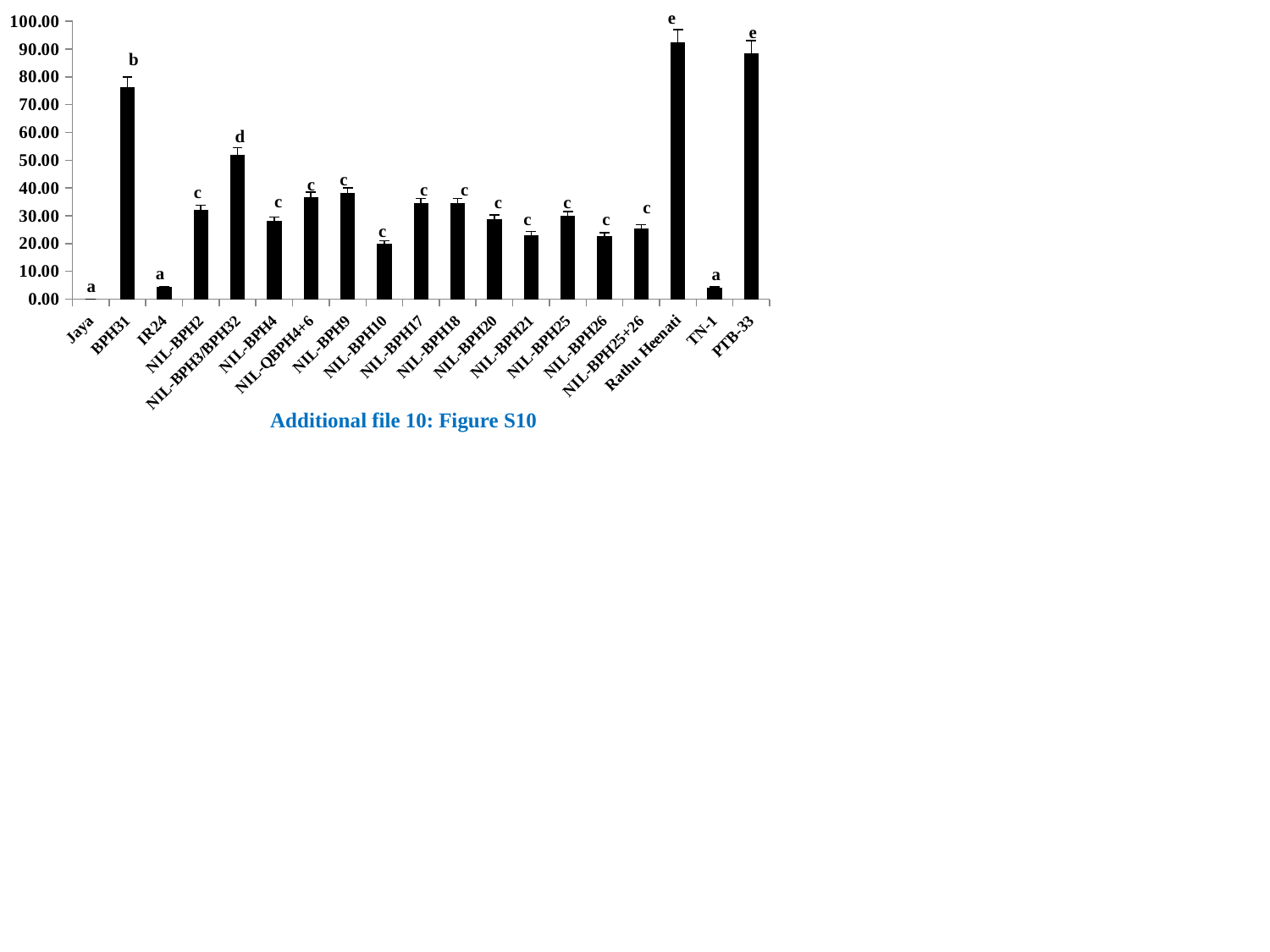

### Chart
| Category | Seedling survival rate |
|---|---|
| Jaya | 0.0 |
| BPH31 | 76.16666666666666 |
| IR24 | 4.2777777777777795 |
| NIL-BPH2 | 32.145 |
| NIL-BPH3/BPH32 | 51.907894736842074 |
| NIL-BPH4 | 28.14142857142857 |
| NIL-QBPH4+6 | 36.66666666666662 |
| NIL-BPH9 | 38.14 |
| NIL-BPH10 | 20.0 |
| NIL-BPH17 | 34.56140350877193 |
| NIL-BPH18 | 34.49833333333335 |
| NIL-BPH20 | 28.8235294117647 |
| NIL-BPH21 | 23.125 |
| NIL-BPH25 | 30.0 |
| NIL-BPH26 | 22.77777777777778 |
| NIL-BPH25+26 | 25.555555555555557 |
| Rathu Heenati | 92.30769230769228 |
| TN-1 | 4.166666666666666 |
| PTB-33 | 88.54489164086687 |e
e
b
c
a
a
d
c
c
c
c
c
c
c
c
c
c
c
a
Additional file 10: Figure S10
