# Supplementary material for: Identification and fine mapping of a new gene, BPH31 conferring resistance to brown planthopper biotype 4 of India to improve rice, Oryza sativa L
Source: Rice (N Y). 2017 Aug 31;10:41. doi: 10.1186/s12284-017-0178-x (PMC5578944; doi:10.1186/s12284-017-0178-x)
Supplement: Additional file 11: Table S1. — List of newly designed InDel markers for fine mapping of BPH31. (PPTX 77 kb) [file 12284_2017_178_MOESM11_ESM.pptx]

## Slide 1
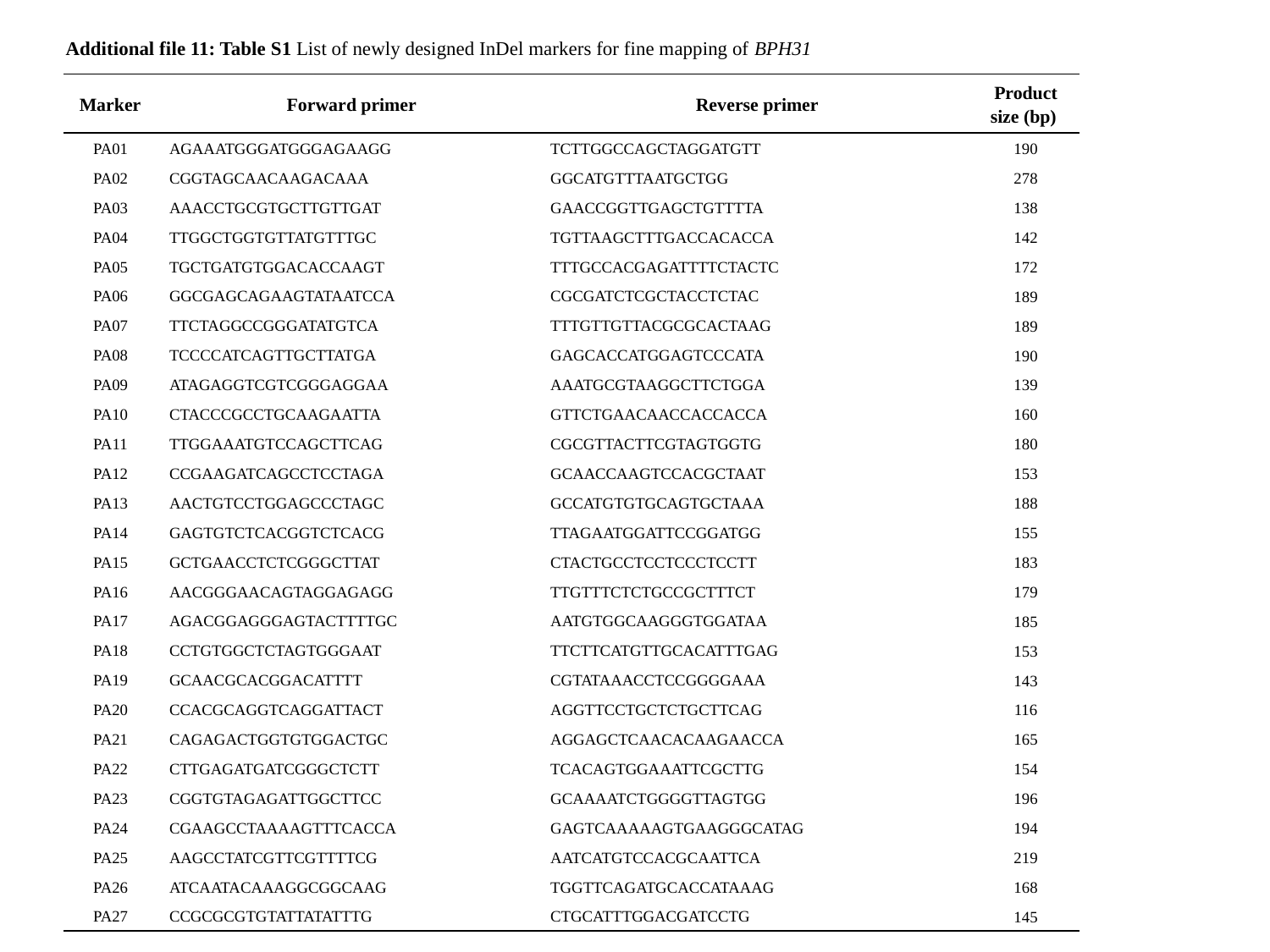

Additional file 11: Table S1 List of newly designed InDel markers for fine mapping of BPH31
| Marker | Forward primer | Reverse primer | Product size (bp) |
| --- | --- | --- | --- |
| PA01 | AGAAATGGGATGGGAGAAGG | TCTTGGCCAGCTAGGATGTT | 190 |
| PA02 | CGGTAGCAACAAGACAAA | GGCATGTTTAATGCTGG | 278 |
| PA03 | AAACCTGCGTGCTTGTTGAT | GAACCGGTTGAGCTGTTTTA | 138 |
| PA04 | TTGGCTGGTGTTATGTTTGC | TGTTAAGCTTTGACCACACCA | 142 |
| PA05 | TGCTGATGTGGACACCAAGT | TTTGCCACGAGATTTTCTACTC | 172 |
| PA06 | GGCGAGCAGAAGTATAATCCA | CGCGATCTCGCTACCTCTAC | 189 |
| PA07 | TTCTAGGCCGGGATATGTCA | TTTGTTGTTACGCGCACTAAG | 189 |
| PA08 | TCCCCATCAGTTGCTTATGA | GAGCACCATGGAGTCCCATA | 190 |
| PA09 | ATAGAGGTCGTCGGGAGGAA | AAATGCGTAAGGCTTCTGGA | 139 |
| PA10 | CTACCCGCCTGCAAGAATTA | GTTCTGAACAACCACCACCA | 160 |
| PA11 | TTGGAAATGTCCAGCTTCAG | CGCGTTACTTCGTAGTGGTG | 180 |
| PA12 | CCGAAGATCAGCCTCCTAGA | GCAACCAAGTCCACGCTAAT | 153 |
| PA13 | AACTGTCCTGGAGCCCTAGC | GCCATGTGTGCAGTGCTAAA | 188 |
| PA14 | GAGTGTCTCACGGTCTCACG | TTAGAATGGATTCCGGATGG | 155 |
| PA15 | GCTGAACCTCTCGGGCTTAT | CTACTGCCTCCTCCCTCCTT | 183 |
| PA16 | AACGGGAACAGTAGGAGAGG | TTGTTTCTCTGCCGCTTTCT | 179 |
| PA17 | AGACGGAGGGAGTACTTTTGC | AATGTGGCAAGGGTGGATAA | 185 |
| PA18 | CCTGTGGCTCTAGTGGGAAT | TTCTTCATGTTGCACATTTGAG | 153 |
| PA19 | GCAACGCACGGACATTTT | CGTATAAACCTCCGGGGAAA | 143 |
| PA20 | CCACGCAGGTCAGGATTACT | AGGTTCCTGCTCTGCTTCAG | 116 |
| PA21 | CAGAGACTGGTGTGGACTGC | AGGAGCTCAACACAAGAACCA | 165 |
| PA22 | CTTGAGATGATCGGGCTCTT | TCACAGTGGAAATTCGCTTG | 154 |
| PA23 | CGGTGTAGAGATTGGCTTCC | GCAAAATCTGGGGTTAGTGG | 196 |
| PA24 | CGAAGCCTAAAAGTTTCACCA | GAGTCAAAAAGTGAAGGGCATAG | 194 |
| PA25 | AAGCCTATCGTTCGTTTTCG | AATCATGTCCACGCAATTCA | 219 |
| PA26 | ATCAATACAAAGGCGGCAAG | TGGTTCAGATGCACCATAAAG | 168 |
| PA27 | CCGCGCGTGTATTATATTTG | CTGCATTTGGACGATCCTG | 145 |
