# Supplementary material for: Identification and fine mapping of a new gene, BPH31 conferring resistance to brown planthopper biotype 4 of India to improve rice, Oryza sativa L
Source: Rice (N Y). 2017 Aug 31;10:41. doi: 10.1186/s12284-017-0178-x (PMC5578944; doi:10.1186/s12284-017-0178-x)
Supplement: Additional file 12: Table S2. — List of candidate genes identified within the fine-mapped region (475 kb) along with their putative function. (PPTX 86 kb) [file 12284_2017_178_MOESM12_ESM.pptx]

## Slide 1
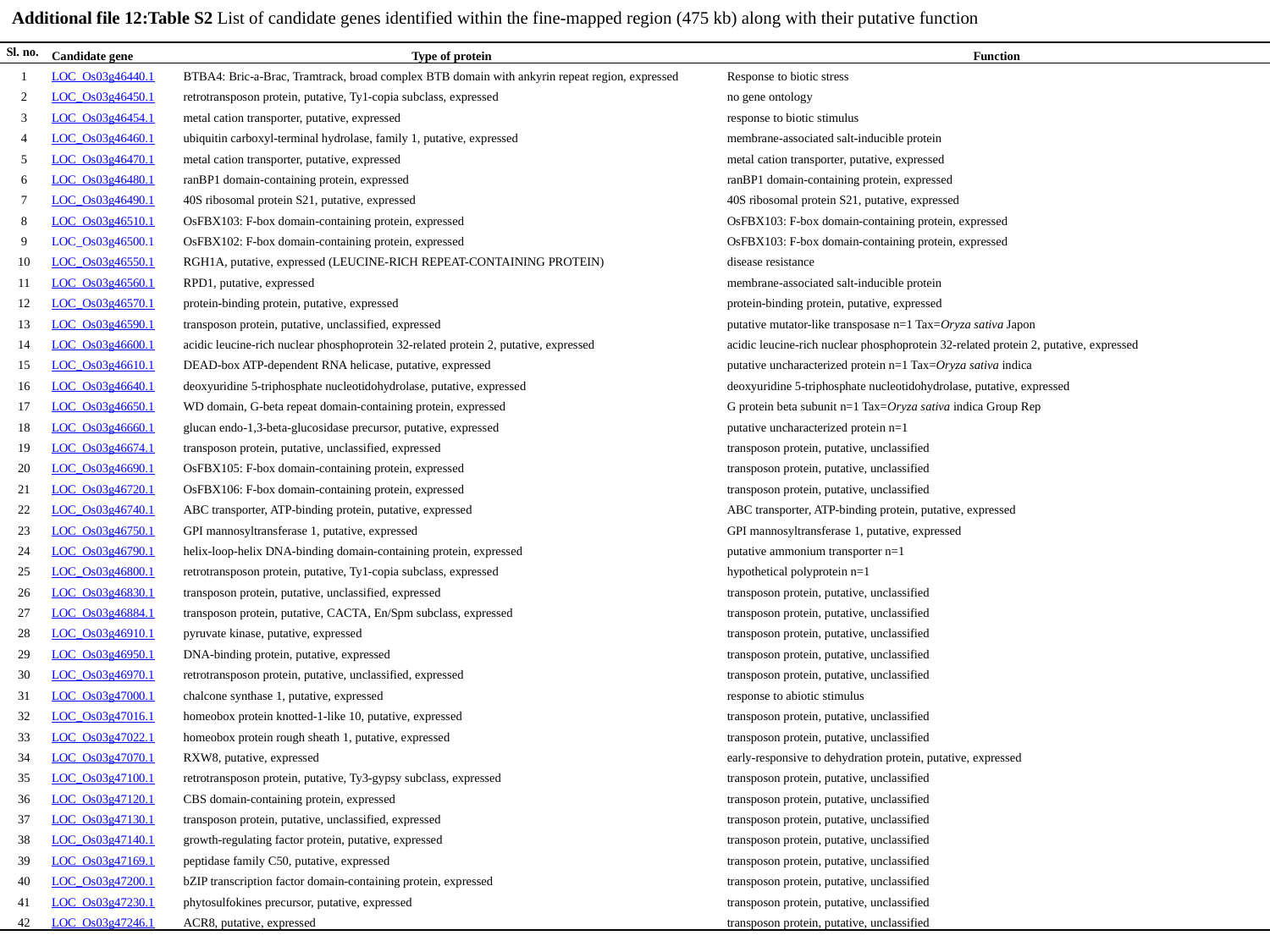

Additional file 12:Table S2 List of candidate genes identified within the fine-mapped region (475 kb) along with their putative function
| Sl. no. | Candidate gene | Type of protein | Function |
| --- | --- | --- | --- |
| 1 | LOC\_Os03g46440.1 | BTBA4: Bric-a-Brac, Tramtrack, broad complex BTB domain with ankyrin repeat region, expressed | Response to biotic stress |
| 2 | LOC\_Os03g46450.1 | retrotransposon protein, putative, Ty1-copia subclass, expressed | no gene ontology |
| 3 | LOC\_Os03g46454.1 | metal cation transporter, putative, expressed | response to biotic stimulus |
| 4 | LOC\_Os03g46460.1 | ubiquitin carboxyl-terminal hydrolase, family 1, putative, expressed | membrane-associated salt-inducible protein |
| 5 | LOC\_Os03g46470.1 | metal cation transporter, putative, expressed | metal cation transporter, putative, expressed |
| 6 | LOC\_Os03g46480.1 | ranBP1 domain-containing protein, expressed | ranBP1 domain-containing protein, expressed |
| 7 | LOC\_Os03g46490.1 | 40S ribosomal protein S21, putative, expressed | 40S ribosomal protein S21, putative, expressed |
| 8 | LOC\_Os03g46510.1 | OsFBX103: F-box domain-containing protein, expressed | OsFBX103: F-box domain-containing protein, expressed |
| 9 | LOC\_Os03g46500.1 | OsFBX102: F-box domain-containing protein, expressed | OsFBX103: F-box domain-containing protein, expressed |
| 10 | LOC\_Os03g46550.1 | RGH1A, putative, expressed (LEUCINE-RICH REPEAT-CONTAINING PROTEIN) | disease resistance |
| 11 | LOC\_Os03g46560.1 | RPD1, putative, expressed | membrane-associated salt-inducible protein |
| 12 | LOC\_Os03g46570.1 | protein-binding protein, putative, expressed | protein-binding protein, putative, expressed |
| 13 | LOC\_Os03g46590.1 | transposon protein, putative, unclassified, expressed | putative mutator-like transposase n=1 Tax=Oryza sativa Japon |
| 14 | LOC\_Os03g46600.1 | acidic leucine-rich nuclear phosphoprotein 32-related protein 2, putative, expressed | acidic leucine-rich nuclear phosphoprotein 32-related protein 2, putative, expressed |
| 15 | LOC\_Os03g46610.1 | DEAD-box ATP-dependent RNA helicase, putative, expressed | putative uncharacterized protein n=1 Tax=Oryza sativa indica |
| 16 | LOC\_Os03g46640.1 | deoxyuridine 5-triphosphate nucleotidohydrolase, putative, expressed | deoxyuridine 5-triphosphate nucleotidohydrolase, putative, expressed |
| 17 | LOC\_Os03g46650.1 | WD domain, G-beta repeat domain-containing protein, expressed | G protein beta subunit n=1 Tax=Oryza sativa indica Group Rep |
| 18 | LOC\_Os03g46660.1 | glucan endo-1,3-beta-glucosidase precursor, putative, expressed | putative uncharacterized protein n=1 |
| 19 | LOC\_Os03g46674.1 | transposon protein, putative, unclassified, expressed | transposon protein, putative, unclassified |
| 20 | LOC\_Os03g46690.1 | OsFBX105: F-box domain-containing protein, expressed | transposon protein, putative, unclassified |
| 21 | LOC\_Os03g46720.1 | OsFBX106: F-box domain-containing protein, expressed | transposon protein, putative, unclassified |
| 22 | LOC\_Os03g46740.1 | ABC transporter, ATP-binding protein, putative, expressed | ABC transporter, ATP-binding protein, putative, expressed |
| 23 | LOC\_Os03g46750.1 | GPI mannosyltransferase 1, putative, expressed | GPI mannosyltransferase 1, putative, expressed |
| 24 | LOC\_Os03g46790.1 | helix-loop-helix DNA-binding domain-containing protein, expressed | putative ammonium transporter n=1 |
| 25 | LOC\_Os03g46800.1 | retrotransposon protein, putative, Ty1-copia subclass, expressed | hypothetical polyprotein n=1 |
| 26 | LOC\_Os03g46830.1 | transposon protein, putative, unclassified, expressed | transposon protein, putative, unclassified |
| 27 | LOC\_Os03g46884.1 | transposon protein, putative, CACTA, En/Spm subclass, expressed | transposon protein, putative, unclassified |
| 28 | LOC\_Os03g46910.1 | pyruvate kinase, putative, expressed | transposon protein, putative, unclassified |
| 29 | LOC\_Os03g46950.1 | DNA-binding protein, putative, expressed | transposon protein, putative, unclassified |
| 30 | LOC\_Os03g46970.1 | retrotransposon protein, putative, unclassified, expressed | transposon protein, putative, unclassified |
| 31 | LOC\_Os03g47000.1 | chalcone synthase 1, putative, expressed | response to abiotic stimulus |
| 32 | LOC\_Os03g47016.1 | homeobox protein knotted-1-like 10, putative, expressed | transposon protein, putative, unclassified |
| 33 | LOC\_Os03g47022.1 | homeobox protein rough sheath 1, putative, expressed | transposon protein, putative, unclassified |
| 34 | LOC\_Os03g47070.1 | RXW8, putative, expressed | early-responsive to dehydration protein, putative, expressed |
| 35 | LOC\_Os03g47100.1 | retrotransposon protein, putative, Ty3-gypsy subclass, expressed | transposon protein, putative, unclassified |
| 36 | LOC\_Os03g47120.1 | CBS domain-containing protein, expressed | transposon protein, putative, unclassified |
| 37 | LOC\_Os03g47130.1 | transposon protein, putative, unclassified, expressed | transposon protein, putative, unclassified |
| 38 | LOC\_Os03g47140.1 | growth-regulating factor protein, putative, expressed | transposon protein, putative, unclassified |
| 39 | LOC\_Os03g47169.1 | peptidase family C50, putative, expressed | transposon protein, putative, unclassified |
| 40 | LOC\_Os03g47200.1 | bZIP transcription factor domain-containing protein, expressed | transposon protein, putative, unclassified |
| 41 | LOC\_Os03g47230.1 | phytosulfokines precursor, putative, expressed | transposon protein, putative, unclassified |
| 42 | LOC\_Os03g47246.1 | ACR8, putative, expressed | transposon protein, putative, unclassified |
