# Supplementary material for: Identification and fine mapping of a new gene, BPH31 conferring resistance to brown planthopper biotype 4 of India to improve rice, Oryza sativa L
Source: Rice (N Y). 2017 Aug 31;10:41. doi: 10.1186/s12284-017-0178-x (PMC5578944; doi:10.1186/s12284-017-0178-x)
Supplement: Additional file 10: Table S3. — Seven years BPH (biotype 4) bioassay result of parental lines and checks. Phenotypic values are the average seedling survival rates. (PPTX 55 kb) [file 12284_2017_178_MOESM13_ESM.pptx]

## Slide 1
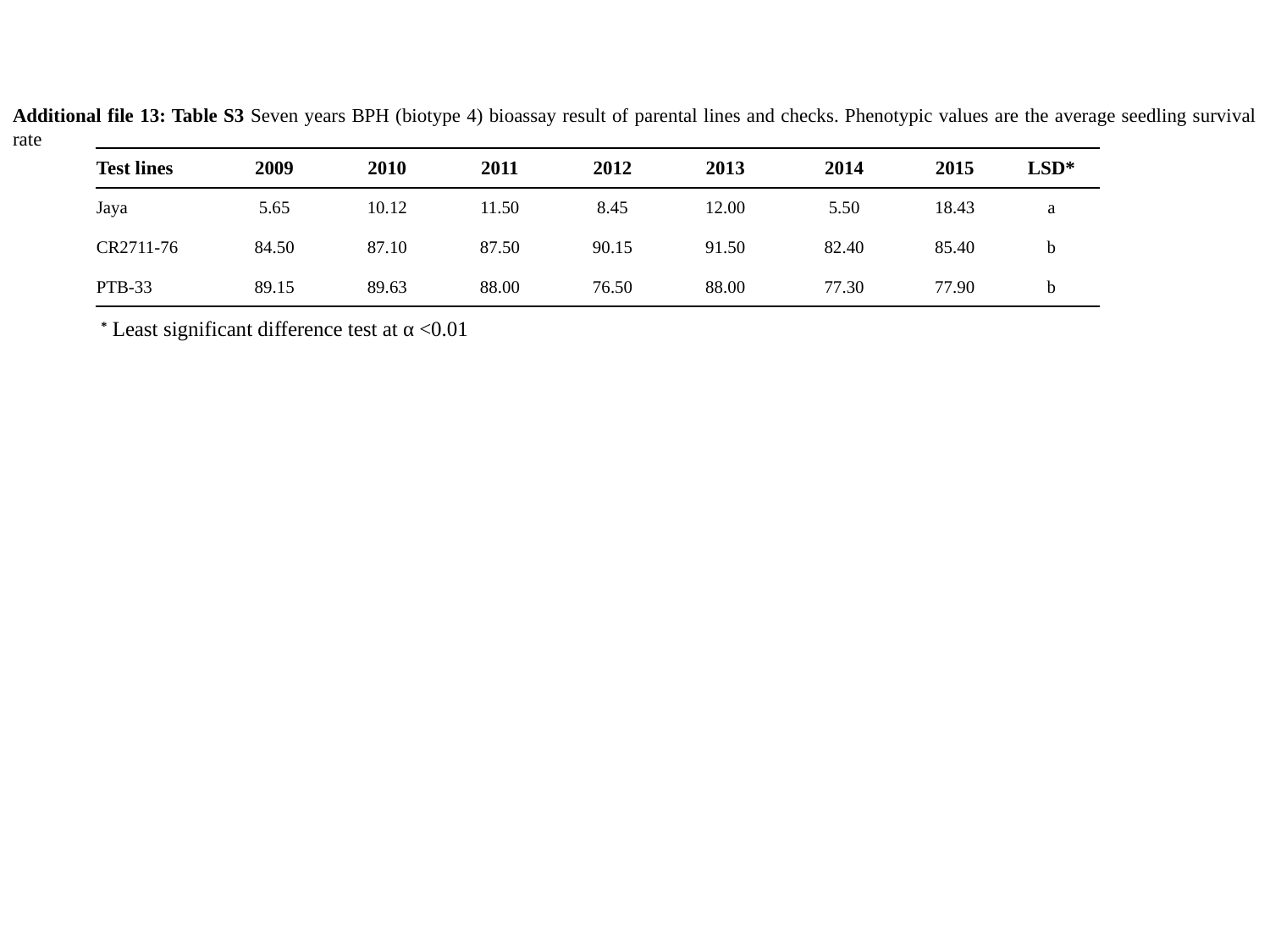

Additional file 13: Table S3 Seven years BPH (biotype 4) bioassay result of parental lines and checks. Phenotypic values are the average seedling survival rate
| Test lines | 2009 | 2010 | 2011 | 2012 | 2013 | 2014 | 2015 | LSD\* |
| --- | --- | --- | --- | --- | --- | --- | --- | --- |
| Jaya | 5.65 | 10.12 | 11.50 | 8.45 | 12.00 | 5.50 | 18.43 | a |
| CR2711-76 | 84.50 | 87.10 | 87.50 | 90.15 | 91.50 | 82.40 | 85.40 | b |
| PTB-33 | 89.15 | 89.63 | 88.00 | 76.50 | 88.00 | 77.30 | 77.90 | b |
* Least significant difference test at α <0.01
